# Bispecific antibodies targeting MPXV A29 and B6 demonstrate efficacy against MPXV infection

**DOI:** 10.1128/jvi.02320-24

**Published:** 2025-04-03

**Authors:** Mengjun Li, Jiayin Chen, Fungxiang Wang, Jiahua Kuang, Yun Peng, Sadia Asghar, Wei Zhao, Yang Yang, Chenguang Shen

**Affiliations:** 1 BSL-3 Laboratory (Guangdong), Guangdong Provincial Key Laboratory of Tropical Disease Research, School of Public Health, Southern Medical University 658728 , Guangzhou, China; 2 National Clinical Research Center for Infectious Disease, Shenzhen Third People’s Hospital, Second Hospital Affiliated to Southern University of Science and Technology 535206 , Shenzhen, China; 3 Department of Laboratory Medicine, Zhujiang Hospital, Southern Medical University 36613, Guangzhou, China; 4 Key Laboratory of Infectious Diseases Research in South China (Southern Medical University), Ministry of Education 70570, Guangzhou, China; 5 State Key Laboratory of Multi-organ Injury Prevention and Treatment, Guangzhou, China; Northwestern University Feinberg School of Medicine, Chicago, Illinois, USA

**Keywords:** MPXV, bispecific antibody, non-competitive epitopes, antiviral activity

## Abstract

Recently, the monkeypox virus (MPXV) outbreak was once again declared by the World Health Organization as a global health emergency, and currently, there is no specific drug against MPXV. During the replication cycle, MPXV produces two distinct forms of viral particles: extracellular enveloped virus (EEV), released via exocytosis, and intracellular mature virus (IMV), expelled through host cell lysis. A29 and B6 proteins are membrane proteins found on the IMV and EEV viral particles, respectively. This study designed two different bispecific antibodies (bsAbs) targeting specific antigens of the MPXV: the developed bsAb 9F8-3A1 targets two non-competitive binding epitopes on the MPXV protein A29, while bsAb 9F8-7C9 targets different antigen-binding epitopes on both A29 and B6. The *in vitro* and *in vivo* characterization assays demonstrated that the bsAbs provided complete protection against three poxvirus strains: vaccinia virus (VACV) Tiantan, VACV Western Reserve (VACV WR), and MPXV, surpassing the efficacy of all the parental monoclonal antibodies. Notably, the bsAb 9F8-7C9 exhibited the most effective antiviral activity. *In vivo* pharmacokinetic experiments showed that these two bsAbs have long half-lives in rhesus macaques. In conclusion, this study successfully developed two bispecific antibodies that target different epitopes, providing crucial insights for the development of decent antiviral drugs against MPXV and other orthopoxviruses.

**IMPORTANCE:**

Mpox is a viral zoonotic disease caused by MPXV infection. Since 2022, cases of mpox have been reported in non-endemic countries. The number of infections and deaths continues to rise, posing a serious threat to global health and safety. Currently, there are no specific treatments for mpox, making the development of effective therapeutic options urgent. In recent years, antibody-based drugs have been extensively studied for the treatment of various significant human viruses. However, there is a lack of research on therapeutic monoclonal antibodies for mpox, particularly in the development and application of bsAbs. In this context, we have designed effective bsAbs that demonstrate high antiviral activity both *in vitro* and *in vivo*. This research provides a theoretical foundation for the development of specific therapeutic agents for mpox and offers new approaches for clinical treatment, which is crucial for controlling the current outbreak.

## INTRODUCTION

Since May 2022, confirmed cases of mpox have been reported in non-endemic countries, with a broad geographic spread and a substantial number of cases (1). According to data from the World Health Organization (WHO), as of 19 December 2024, mpox has been identified in 127 countries and regions worldwide, with over 117,000 reported infections and 263 fatalities (2). The number of cases and deaths continues to rise, with a particularly severe outbreak occurring recently in Africa. Mpox is a zoonotic infection caused by the monkeypox virus (MPXV), an orthopoxvirus (OPXV) that is part of the poxvirus family (3, 4). Transmission of mpox can occur from person to person through contact with infected skin, bodily fluids, or contaminated objects like bedding (5). Typical symptoms of the infection comprise fever, myalgia, swollen lymph nodes, as well as skin and mucous membrane rashes. Severe infections with MPXV can result in mortality (6, 7). Historically, infections from clade I and clade IIb exhibit similar clinical symptoms. However, clade I infections are associated with a fatality rate of approximately 10%, whereas clade IIb infections have a mortality rate of less than 1% (8, 9). The global outbreak of monkeypox, which began in May 2022, has primarily been attributed to clade IIb, with the majority of cases occurring among men and exhibiting mild symptoms. However, by the end of 2023, clade I was prevalent in the Democratic Republic of the Congo and other regions of Central Africa. As of early 2024, more than half of monkeypox cases were reported in children under 15 years of age, with a mortality risk exceeding 10%. In response to the escalating situation, the WHO has once again declared the outbreak a public health emergency of international concern (10–12).

MPXV has two forms, the extracellular enveloped virus (EEV) and the intracellular mature virus (IMV), both of which are infectious. The primary distinction between IMV and EEV lies in the lack of an outermost layer in IMV. IMV, being highly stable, is thought to facilitate host-to-host transmission, whereas EEV has a fragile outer membrane facilitating transmission between cells of the same host (13, 14). Vaccinia virus (VACV) protein A27 is a homologous trimeric extracellular protein that binds to the intracellular mature virion membrane through its C-terminal region and the transmembrane protein A17 (15). VACV protein L1, a myristylated transmembrane protein, is found on the surface of poxviruses within mature virions (16). Both A27 and L1 are direct homologs of the MPXV proteins A29 and M1, respectively, and they play critical roles in virus attachment and entry (17, 18). Additionally, the B6 protein present in the EEV membrane exhibits homology with VACV protein B5. Studies have demonstrated that B5 is vital for multiple stages of viral infection, including the integrity of the EEV envelope, actin tail formation, maintenance of normal plaque size, and virulence. Furthermore, B5 is an important component of the antiviral response against EEV and contributes to protective immunity (19–21). Both A27 and B5 have been identified as targets for neutralizing antibodies, demonstrating their ability to elicit neutralizing antibodies and exhibit potent antiviral effects in both animal models and humans (22–24). MPXV is an enveloped double-stranded DNA virus that shares significant homology with OPXV such as the variola virus, displaying a complex structure. However, unlike other DNA viruses, the currently identified strains of MPXV are rapidly evolving and undergoing continuous mutations (25). Furthermore, as of now, there are no specific therapeutic agents for mpox, highlighting the urgent need for the development of more effective antiviral treatments.

Neutralizing antibodies have been shown to provide effective protection against various viral infections, including SARS-CoV-2, EBV, influenza virus, and HIV-1 (26–29). However, since the two binding regions of the neutralizing monoclonal antibodies (mAbs) target the same epitope, single-antibody therapies can quickly give rise to drug resistance and immune escape, regardless of the neutralization potency and epitope conservation (30). In previous studies, we discovered specific neutralizing monoclonal antibodies targeting MPXV A29, B6, and M1R, namely, A9F8, A3A1, B7C9, M1H11, and M3B2, which efficiently treat poxvirus infections. Notably, the cocktail therapy involving A29 and M1R neutralizing antibodies demonstrated the best antiviral effects both *in vitro* and *in vivo* (31, 32). While cocktail therapies can mitigate antibody resistance, they also increase production costs. Additionally, studies have shown that multiple antibodies have significantly or completely lost their neutralizing activity against the Omicron variant, leading the Food and Drug Administration to restrict the use of two approved antibody cocktails (33). Importantly, bispecific antibodies (bsAbs) represent an emerging therapeutic modality that can simultaneously bind to two different epitopes (34). This improves the neutralization potency and breadth of antibodies while reducing immune escape. Currently, they have become powerful tools in cancer immunotherapy and pathogen prevention, with two bsAbs already approved for clinical use (34, 35). Significantly, there are few reports on the development of bsAbs for the treatment of monkeypox, prompting us to design bsAbs targeting mpox to provide a scientific reference for clinical treatment options.

In our preliminary work, we developed a series of efficient antiviral monoclonal antibodies targeting A29, B6, and M1R. Based on the results of cocktail therapy, we designed and developed a bispecific antibody, Bis-M1M3(32), targeting different binding epitopes of MPXV M1R, which exhibited high antiviral activity both *in vitro* and *in vivo*. Building on this, to explore whether bispecific antibodies targeting other antigen epitopes exhibit similarly high or even greater antiviral efficacy, we further developed bispecific antibodies targeting different epitopes of MPXV A29, as well as another that targets distinct antigen epitopes of both MPXV A29 and MPXV B6. Our research found that these bispecific antibodies demonstrated even higher antiviral activity *in vitro* and showed equally remarkable efficacy in mouse models infected with three strains: VACV Tiantan, VACV Western Reserve (VACV WR), and MPXV.

## RESULTS

### Design and characterization of BsAbs

**Fig 1:**
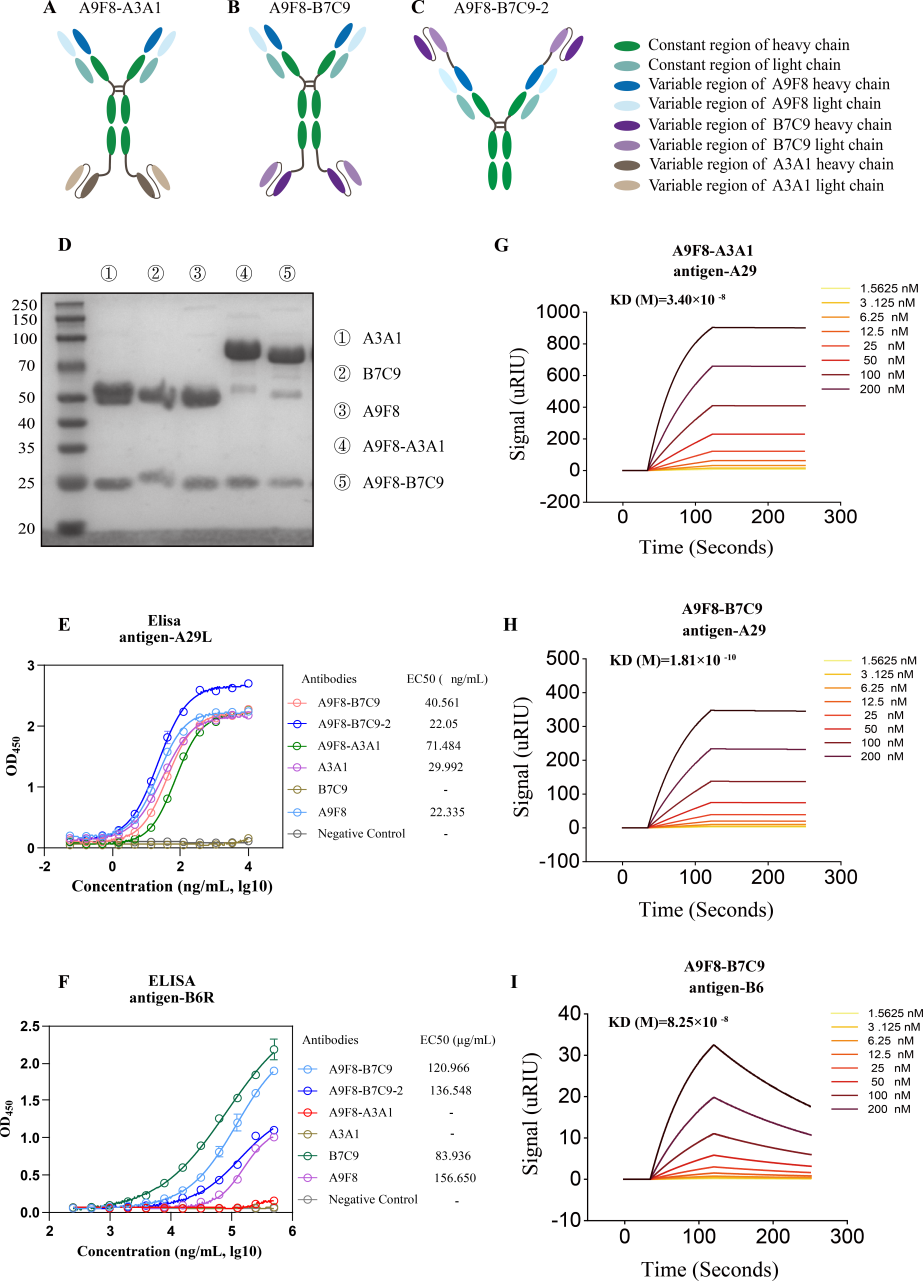
Design and characterization of bsAbs. (**A–C**) Formats of the bsAbs designed. Each antibody color represents a different structure (green, constant region of the heavy chain; light green, constant region of light chain; dark blue, A9F8 heavy-chain variable region; light blue, A9F8 light-chain variable region; brown, A3A1 ScFv structure; purple, B7C9 ScFv structure). (**D**) The reducing SDS-PAGE analysis of the purified bsAbs from the 293F expression system. (**E and F**) The binding activities of A3A1, B7C9, A9F8, and the designed bsAbs to MPXV A29 and MPXV B6 were tested by ELISA. Data are expressed by means ± SEM (*n* = 3). (**G–I**) Binding affinity analysis of bsAbs to MPXV A29 and MPXV B6.

According to previously published results, the neutralizing antibodies A9F8 and A3A1 targeting A29 exhibit strong antiviral activity both *in vitro* and *in vivo*. Additionally, the neutralizing antibody B7C9, which targets B6, has been identified and validated for its effective binding and neutralization of the vaccinia virus *in vitro* (31). Based on these antibodies, two distinct symmetrical forms of IgG-ScFv were designed. bsAb A9F8-B7C9 contains the A9F8 heavy-chain (HC)/light-chain (LC) variable region, the human IgG1 Fc region, human kappa LC, and B7C9 ScFv structure. The bsAb A9F8-A3A1 contains the A9F8 heavy-chain/light-chain variable region, the human IgG1 Fc region, and the ScFv structure of A3A1 (Fig. 1A through C). To verify the influence of the Fc segment on the stability and neutralization activity of dual resistance, the ScFv structure of 7C9 in bsAb A9F8-B7C9 was connected to the CDR region of 9F8 and the human IgG1 Fc region through (G4S)3, respectively. In addition, to better compare bsAbs with other monoclonal antibodies, we also constructed human-derived chimeric monoclonal antibodies A3A1, B7C9, and A9F8. These engineered bsAbs and humanized monoclonal antibodies were expressed using the 293F expression system and purified to a high purity (Fig. 1D). Enzyme-linked immunosorbent assay (ELISA) was used to preliminarily determine the binding of bsAbs and humanized monoclonal antibodies to proteins. As shown in Fig. 1E and F, it was found that bsAb A9F8-B7C9-1 and bsAb A9F8-B7C9-2 had strong binding capacity with A29. The EC50 was less than 50 ng/mL and showed cross-binding ability, and the EC50 values of binding with B6 were 120.966 and 136.548 ng/mL, respectively. However, bsAb A9F8-A3A1 only has strong binding with A29, and the EC50 value is 71.484 ng/mL. A3A1, A9F8, and A29 have strong binding, and EC50 values are less than 30 ng/mL. B7C9 and B6 showed strong binding ability, with an EC50 value of 83.936 ng/mL. However, due to the low yield, we could not conduct further in-depth study on bsAb A9F8-B7C9-2. Additionally, affinity measurements using surface plasmon resonance (SPR) confirmed that the bsAb A9F8-B7C9 (a shorthand for A9F8-B7C9-1) demonstrated similar affinity for A29 or B6 when compared to A3A1, A9F8, or B7C9. In contrast to the single binding of monoclonal antibodies, the bsAb A9F8-B7C9 exhibited high affinity for both A29 and B6 by targeting different antigenic epitopes. This suggests that the bsAb may provide broader protective effects (Fig. 1G through I; Fig. S1).

### bsAbs effectively neutralize the virus

**Fig 2:**
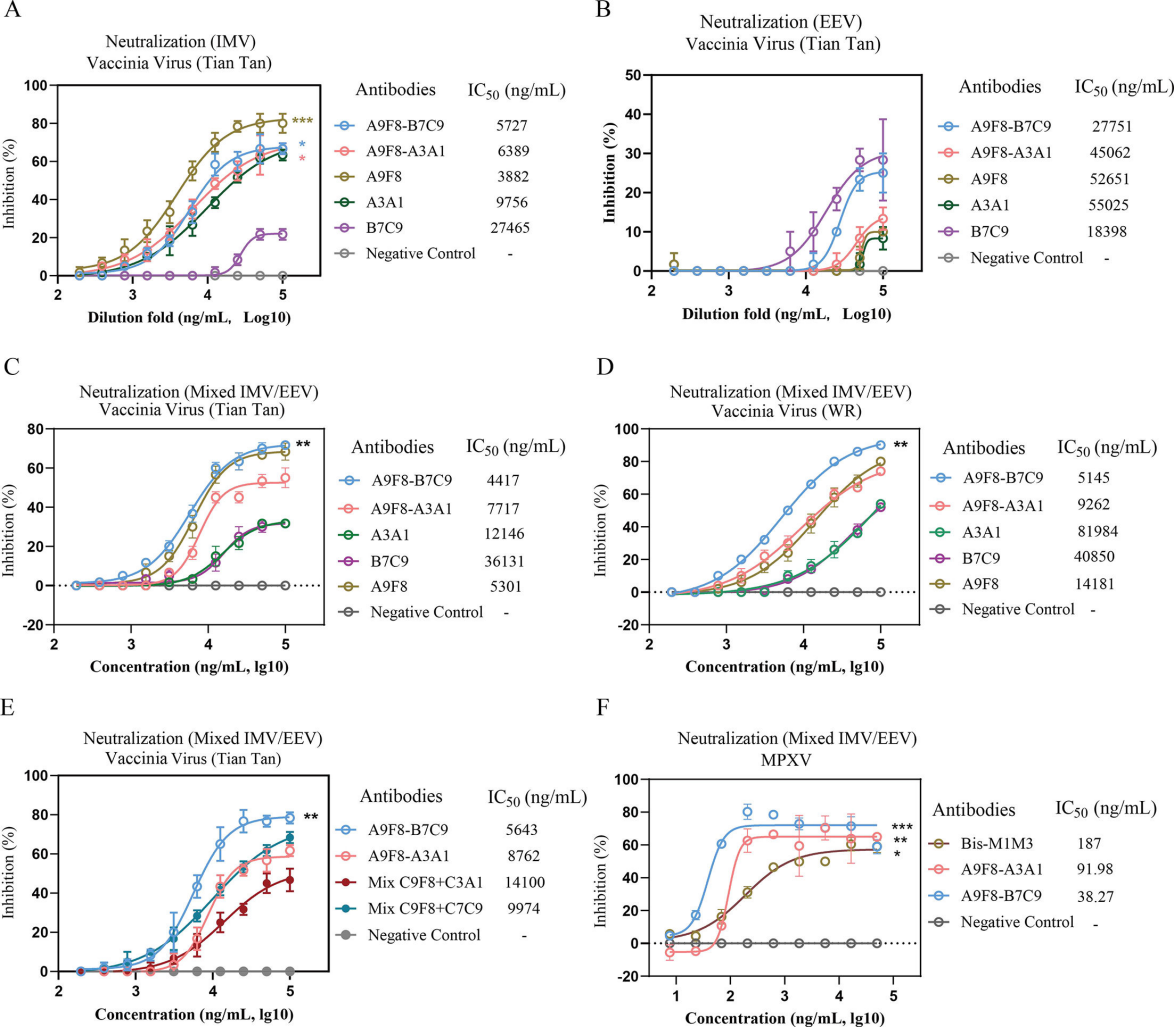
Complement-dependent neutralizing antibodies against VACV Tiantan, VACV WR, and MPXV. (**A and B**) Three mAbs and two bsAbs were analyzed using the VACV Tiantan IMV (**A**) and EEV (**B**). (**C and D**) Three mAbs and two bsAbs were analyzed using the VACV Tiantan (**C**) and VACV WR (**D**) (*n*  =  3). (**E**) “Cocktail” therapy, mixtures of A9F8 with A3A1 and A9F8 with B7C9 (*n* = 3). (**F**) Three bsAbs were analyzed using MPXV (*n*  =  3). The IC_50_ was determined by log (inhibitor) response of nonlinear regression and is displayed as the mean ± SEM. Statistical significance was calculated via ordinary one-way analysis of variance. *P < 0.05, **P < 0.01, ***P < 0.001.

It has been reported that anti-EEV monoclonal antibodies exert their protective function only in the presence of a complement (20). To determine whether the anti-A29 monoclonal antibodies A9F8 and A3A1 share a common neutralization mechanism, fresh EEV and IMV were purified following the previously described protocol (20). Neutralization assays against IMV were conducted in both the presence and absence of a complement. As illustrated in Fig. S2A, the presence of a complement significantly enhanced the neutralizing activity of both antibodies, with A9F8 and A3A1 exhibiting IC50 values of 7,032 and 15,552 ng/mL, respectively. These findings demonstrate that a complement is essential for the neutralization activity of both monoclonal antibodies. Furthermore, the neutralizing efficacy of each monoclonal antibody against EEV was evaluated under identical experimental conditions. Consistent with our expectations, neither A9F8 nor A3A1 exhibited neutralizing activity against EEV, regardless of complement presence. In contrast, the anti-B6 monoclonal antibody B7C9 required a complement to mediate viral neutralization (Fig. S2B). Collectively, these results indicate that the neutralizing activities of these monoclonal antibodies are complement dependent. Furthermore, to ascertain the neutralizing activities of each antibody against IMV and EEV, subsequent neutralization experiments were carried out. As shown in Fig. 2A and B, it was observed that all antibodies demonstrated varying degrees of neutralization. A9F8-B7C9 exhibited potent neutralizing activities against both IMV and EEV, with IC_50_ values of 5,727 and 27,751 ng/mL, respectively. In contrast, with complement, anti-A29 monoclonal antibodies A9F8 and A3A1 only displayed robust neutralizing activities against IMV, with IC_50_ values of 3,882 and 9,756 ng/mL, respectively. The B6 monoclonal antibody B7C9 exhibited weak neutralizing activities against both IMV and EEV. Given that B6 is an EEV-specific protein, to more accurately ascertain the *in vitro* neutralizing activity of the anti-B6 monoclonal antibody B7C9, we employed the comet inhibition assay for identification. This assay quantifies the inhibition of the EEV form released by the virus on the formation of comet-like plaques (36). As depicted in Fig. S3, in the absence of antibody, the EEV of VACV Tiantan generated comet-like patches. However, in the B7C9 treatment group, the formation of comets was markedly reduced, while in the bispecific antibody A9F8-B7C9 treatment group, a slight reduction was noted. Such an effect was absent in the other groups. These findings suggest that both antibodies (B7C9 and A9F8-B7C9) could, to varying extents, impede the formation and release of EEV.

To further characterize the neutralizing activity of the aforementioned antibodies, a neutralization assay was conducted using mixed viruses of VACV Tiantan, VACV WR, and MPXV strains. As depicted in Fig. 2C and D, bsAb A9F8-B7C9 demonstrated potent neutralizing efficacy against VACV Tiantan and VACV WR, with IC_50_ values of 4,417 and 5,145 ng/mL, respectively. In comparison, bsAb A9F8-A3A1 exhibited IC_50_ values of 7,717 and 9,262 ng/mL for VACV Tiantan and VACV WR, respectively. Notably, bsAb A9F8-B7C9 possessed lower IC_50_ values than bsAb A9F8-A3A1, suggesting superior *in vitro* neutralizing potency. Furthermore, the IC_50_ values for A9F8, A3A1, and B7C9 were all elevated relative to the two bsAbs, signifying that both bsAb A9F8-B7C9 and bsAb A9F8-A3A1 not only preserved the neutralizing capabilities of their parental antibodies but also enhanced the neutralizing efficacy. Simultaneously, the *in vitro* neutralization of VACV Tiantan was validated via the combination of monoclonal antibodies using “cocktail” therapy (Fig. 2E). It was observed that the mixtures of A9F8 with A3A1, as well as A9F8 with B7C9, exhibited higher IC_50_ values, specifically 14,100 and 9,974 ng/mL, respectively. Although they also exerted a certain neutralizing effect *in vitro*, in adherence to the cost-effectiveness principle, the superiority of bispecific antibodies was more prominent. Additionally, to ascertain the *in vitro* anti-MPXV activities of bsAb A9F8-A3A1 and bsAb A9F8-B7C9, a comparative analysis with Bis-M1M3 was conducted, as presented in Fig. 2F. The IC_50_ values for bsAb A9F8-B7C9, bsAb A9F8-A3A1, and Bis-M1M3 were determined to be 38.27, 91.98, and 187 ng/mL, respectively. These findings indicate that all three antibodies exhibited robust *in vitro* neutralizing activity, with bsAb A9F8-B7C9 demonstrating the most pronounced effect.

**Fig 3:**
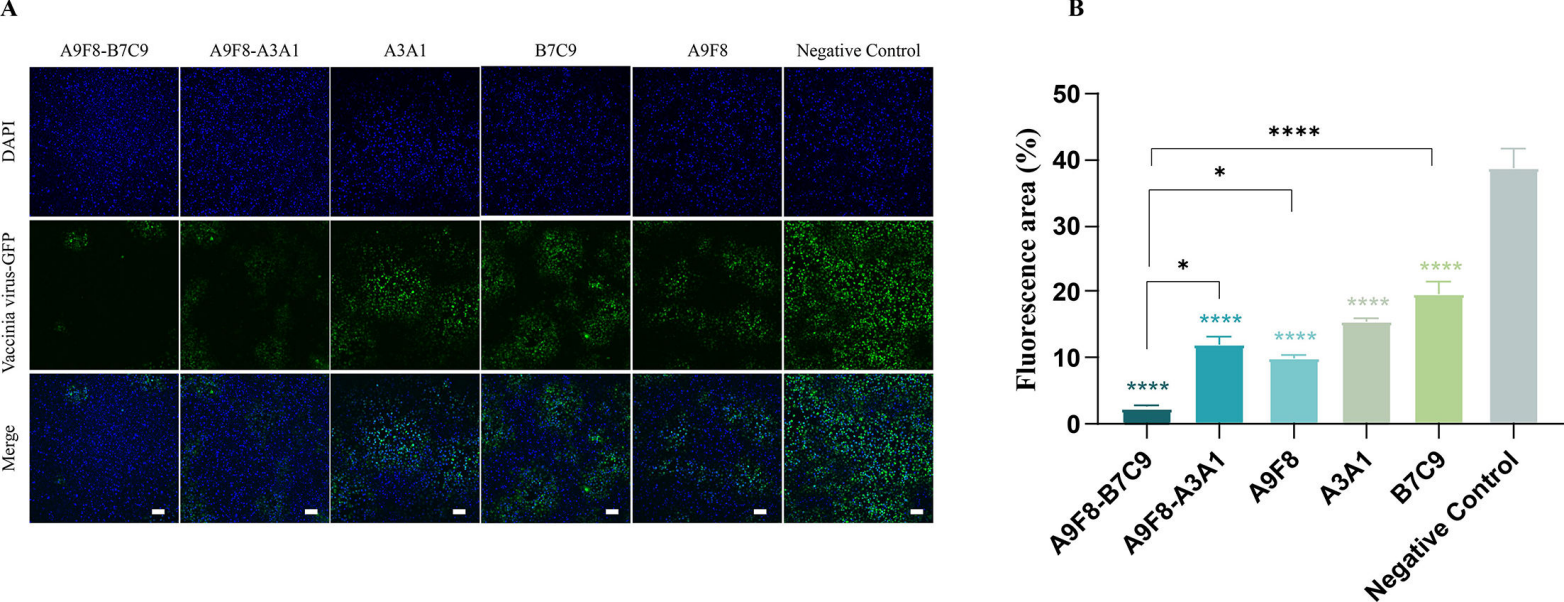
Confocal images of mAb and bsAb immunofluorescence experiments. (**A**) This was conducted using a viral mixture of VACV (Tiantan) EEV and mature virion. The VACV Tiantan was labeled with quantum dots (green fluorescent protein, green), and nucleus (DAPI, blue). Groups are conducted in 5% of the presence of complement. Scale bars, 100 µm. (**B**) Quantitative analysis of fluorescence area in different treatment groups using ImageJ software. Data are displayed as mean ± SEM (*n* = 3). Statistical significance was calculated via ordinary one-way analysis of variance. *P < 0.05, ****P < 0.001.

Furthermore, to directly assess the neutralizing activity of each antibody *in vitro*, we conducted an immunofluorescence experiment by incubating an equal volume of antibody with a concentration of 0.05 mg/mL along with 100 TCID_50_ VACV (Tiantan) for infecting cells. The findings are illustrated in Fig. 3A. The viral quantities of bsAbs A9F8-B7C9 and A9F8-A3A1 were both lower than those of the individual monoclonal antibodies. Quantitative statistical analysis (Fig. 3B) indicated that the bsAbs exhibited superior anti-VACV activity *in vitro* compared to the monoclonal antibodies, with A9F8-B7C9 demonstrating the most effective results, consistent with the aforementioned findings. Conversely, the cells were initially infected with the virus and subsequently incubated with an equal volume of antibody (Fig. S4A and B). Quantitative statistical analysis revealed that there was no significant difference between the various antibody groups and the negative control group. This finding indicates that the antibody fails to exert its effect once the virus has entered the cells.

### bsAbs protect against VACV (Tiantan), VACV WR, and MPXV infection *in vivo*

**Fig 4:**
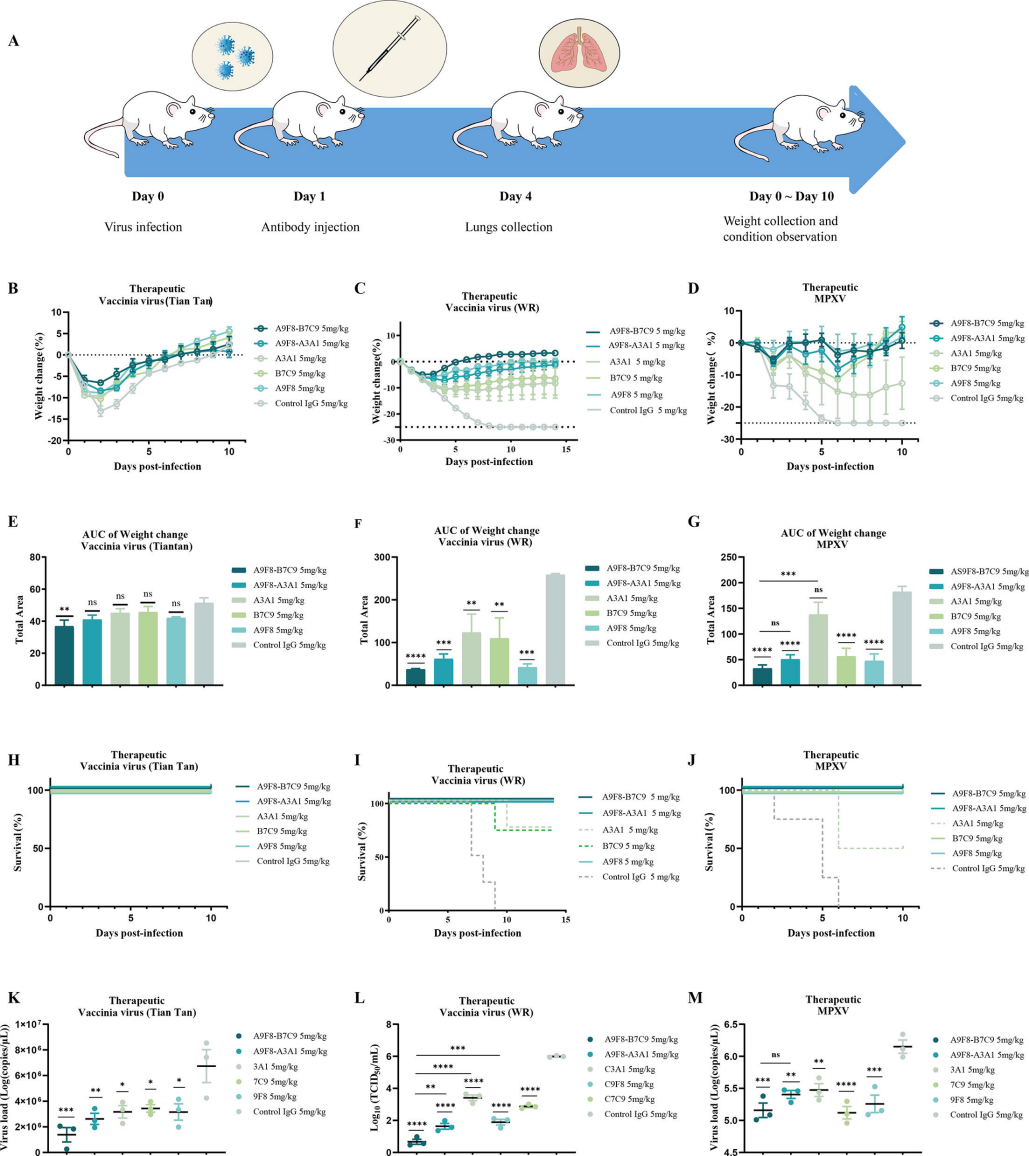
Therapeutic efficacy of bsAbs in a mouse model of VACV (Tiantan), VACV WR, and MPXV infections. (**A**) Schematic diagram of infection model and treatment in mice. (**B–D**) Rate of weight change in mice infected with VACV (Tiantan), VACV WR, and MPXV, respectively. (**E–G**) The area under the curve (AUC) of the weight change rate of mice over time. (**H–J**) Survival curves of mice infected with VACV (Tiantan), VACV WR, and MPXV. (**K–M**) Pulmonary virus titer was detected by reverse transcription PCR. All data are shown as means with SEM and were analyzed by one-way analysis of variance. Statistical significance was calculated via ordinary one-way analysis of variance. *P < 0.05, **P < 0.01, ***P < 0.001, ****P < 0.001. ns, not significant.

To further assess the protective effects of bsAbs A9F8-B7C9 and A9F8-A3A1, we conducted protective experiments using a mouse model infected with orthopoxvirus. Three strains of VACV (Tiantan), VACV WR, and MPXV were administered intranasally to evaluate the efficacy of the antibodies against different viral infections. As illustrated in Fig. 4A, each antibody (5 mg/kg) was administered intraperitoneally 1 day post-infection, and the mice were euthanized on the fourth day. The lungs of mice in each treatment group were harvested for subsequent viral titer determination and pathological analysis. Additionally, changes in body weight and the overall health status of the mice were monitored and documented for a consecutive period of 10/14 days.

Various antibodies exhibited different levels of antiviral efficacy *in vivo*. Initially, all groups showed a slight decline in body weight and survival rates in response to the VACV Tiantan strain challenge. The area under the curve of body weight and time change in mice treated with bsAb A9F8-B7C9 was the smallest, indicating prompt weight recovery in this cohort with no mortality observed, thereby demonstrating complete protection (Fig. 4B, E, and H). Subsequently, during VACV WR strain and MPXV challenges, more pronounced weight loss was observed across all groups (Fig. 4C, D, F, and G). Notably, varying numbers of A3A1, B7C9, and IgG control group mice succumbed to the VACV WR and MPXV infections, suggesting incomplete protection by these antibodies. However, in these challenges, no mortality was recorded in the three other groups (Fig. 4D and J), with mice treated with A9F8-B7C9 showcasing the swiftest recovery and maximal weight gain. On the other hand, examining pulmonary virus titers revealed significantly lower levels in all treatment groups compared to the control IgG group on the fourth day post-infection with VACV Tiantan (Fig. 4K). Simultaneously, other tissues, namely, the heart, liver, spleen, kidney, ovary, and skin, were harvested for virus detection (Fig. S5). In comparison with the control IgG group, no significant difference was observed in the CT values of the ovaries among the treatment groups, with all values exceeding 35. This suggested that the viral load in the ovaries of each group was relatively low, rendering it ineffective to ascertain whether the antibodies could efficiently impede the *in vivo* dissemination of VACV Tiantan. Moreover, the CT values of all tissues in the control IgG group were also above 35, signifying that only a minimal amount of virus was present in these tissues (37). Notably, in the context of the VACV WR challenge, the bsAb group demonstrated a more antiviral effect than the mAb group, with bsAb A9F8-B7C9 exhibiting superior therapeutic outcomes over A9F8-A3A1 (Fig. 4L). Moreover, though all treatment groups displayed a significant reduction in MPXV lung viral titers compared to the control IgG group, no notable variations in titer reductions were observed among the bsAbs (Fig. 4M).

**Fig 5:**
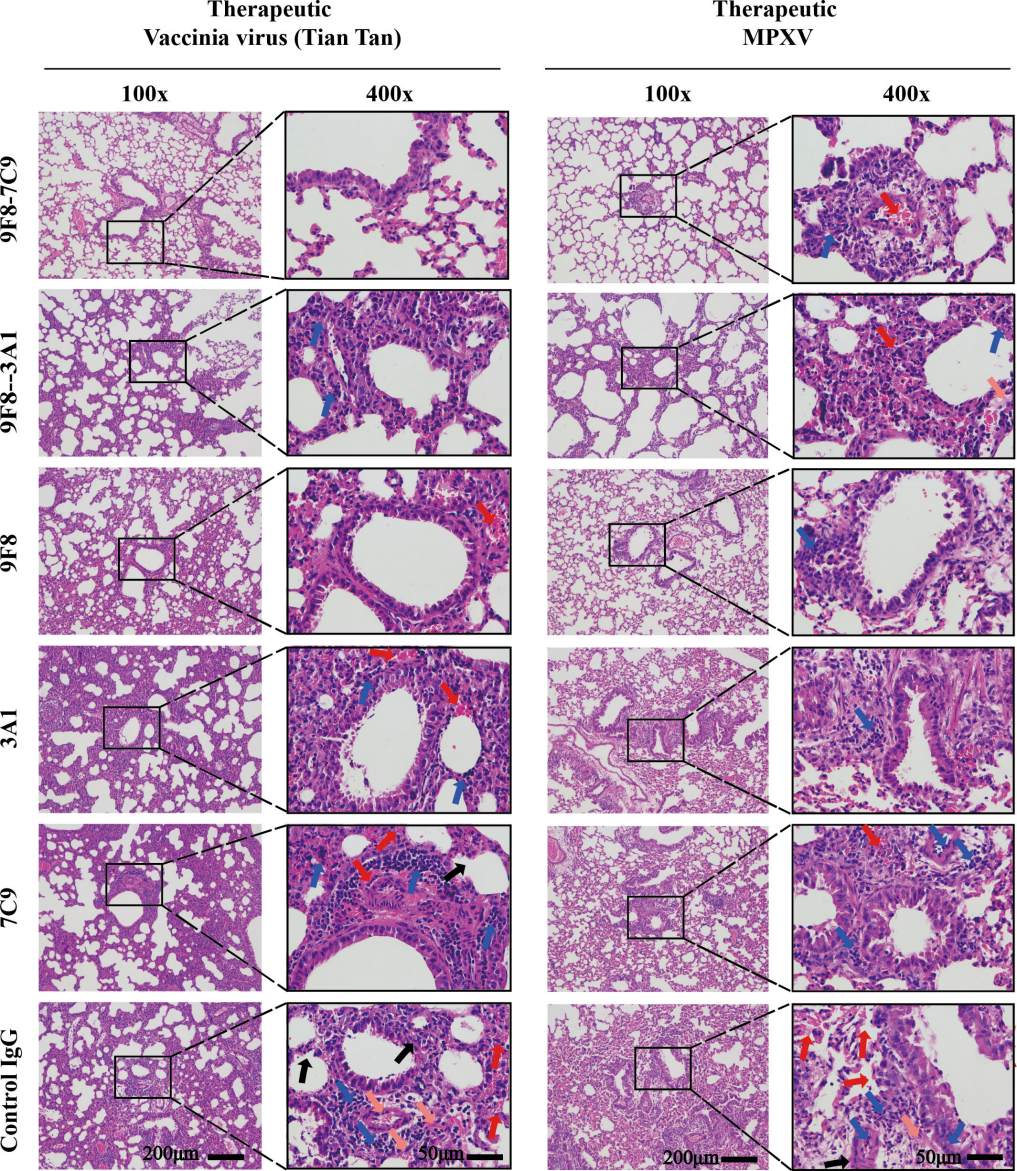
Samples were stored in formalin for 48 h and then embedded in paraffin. Sectioned samples were stained with hematoxylin and eosin. In the control IgG group, the lungs showed alveolar septa thickening (black arrows), multifocal bleeding (red arrows), accumulation of inflammatory cells (including lymphocytes, macrophages, and neutrophils [blue arrows]), and pink infiltration in some alveolar cavities (pink arrows). Pulmonary-specific thickening of alveolar septa (black arrows), multifocal bleeding (red arrows), and accumulation of inflammatory cells (including lymphocytes, macrophages, and neutrophils [blue arrows]) were also seen in the monoclonal antibody group, but symptoms were less severe than those in the control group. Bleeding (red arrows) and accumulation of inflammatory cells (including lymphocytes, macrophages, and neutrophils [blue arrows]) were also seen in the lungs in the dual antibody group, but symptoms were least severe. Scale bars are labeled on the panel.

Histopathological analysis of the lungs of infected animals was conducted subsequently, and the findings are presented in Fig. 5. Observations revealed varying degrees of lesions in the lung tissue of mice across different experimental groups on the fourth day post-infection. In the control IgG group, thickening of the alveolar septum, multifocal hemorrhaging, and accumulation of immune cells (comprising lymphocytes, macrophages, and neutrophils) were observed, along with pink exudates in some alveolar lumens. Similar findings of pulmonary alveolar septum thickening, multifocal hemorrhaging, and immune cell accumulation were noted in the A3A1 and B7C9 groups; however, the severity of symptoms was comparatively milder than that in the control group. The bsAb group exhibited minimal lung injury, with no significant pathological alterations observed.

**Fig 6:**
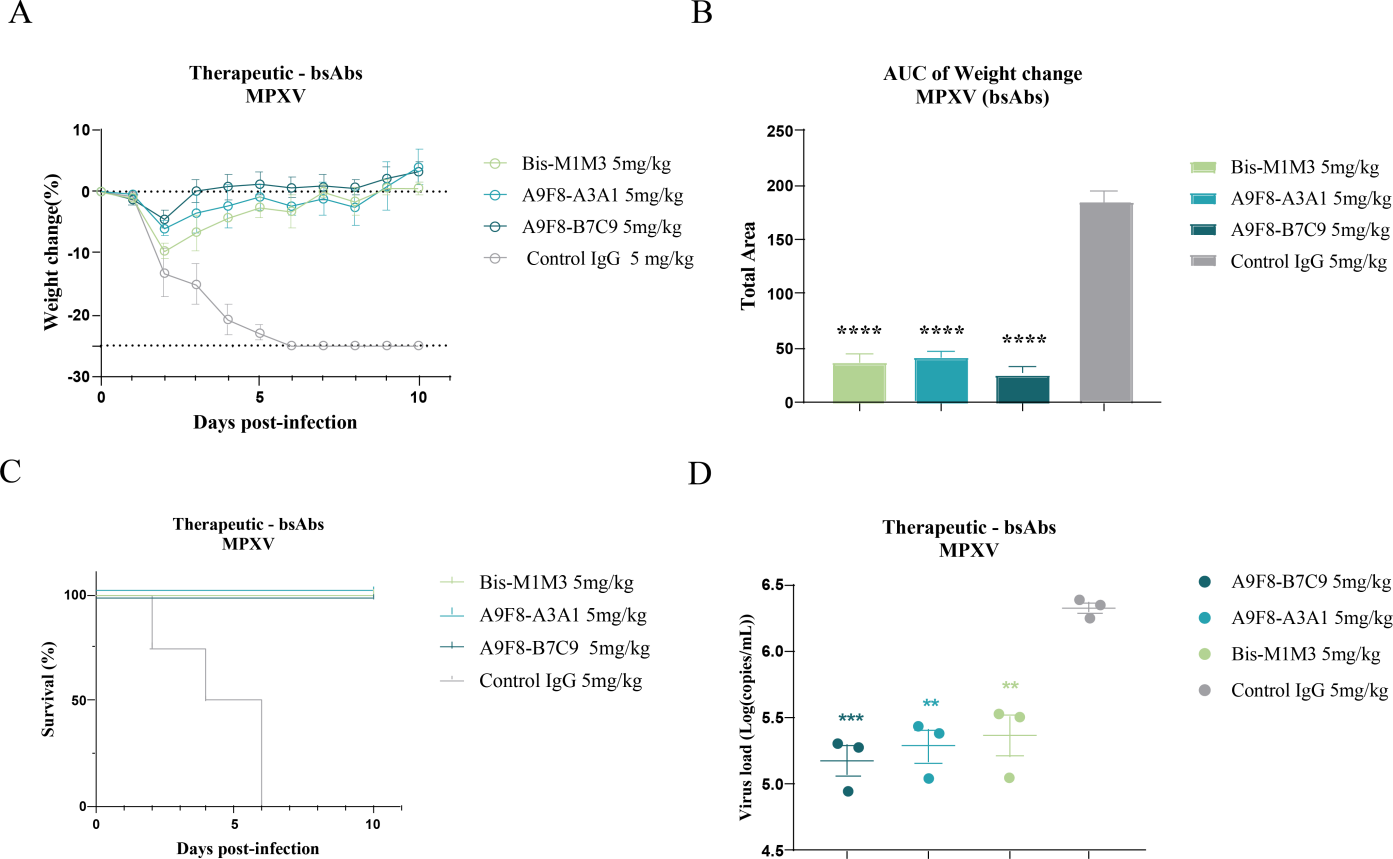
Therapeutic efficacy of bsAbs (Bis-M1M3, bsAb A9F8-A3A1, and bsAb A9F8-B7C9) in a mouse model of MPXV infection. (**A**) Rate of weight change in mice infected with MPXV. (**B**) The area under the curve (AUC) of the weight change rate of mice over time. (**C**) Survival curves of mice infected with MPXV. (**D**) Pulmonary virus titer was detected by reverse transcription PCR. All data are shown as means with SEM and were analyzed by one-way analysis of variance. Statistical significance was calculated via ordinary one-way analysis of variance. **P < 0.01, ***P < 0.001.

Additionally, we evaluated the *in vivo* efficacy of Bis-M1M3, bsAb A9F8-B7C9, and bsAb A9F8-A3A1 against MPXV infection to determine whether bsAb A9F8-B7C9 and bsAb A9F8-A3A1 exhibit similar or superior antiviral activities compared to Bis-M1M3. After the mice were infected with MPXV via the nasal route, they were administered Bis-M1M3, bsAb A9F8-B7C9, or bsAb A9F8-A3A1 (5 mg/kg) via intraperitoneal injection after 24 h. The mice were randomly euthanized on day 4, and lung tissues were collected. Their body weights and health status were continuously monitored and recorded for 10 days. We observed that, compared to the control group, all bsAbs led to varying degrees of weight loss; however, none resulted in mortality, indicating complete protection. Notably, in comparison to the Bis-M1M3 treatment group, the bsAb A9F8-B7C9 and bsAb A9F8-A3A1 treatment groups exhibited less weight loss and a quicker recovery to normal levels, with the bsAb A9F8-B7C9 group demonstrating the fastest recovery (Fig. 6A through C), suggesting a more effective capacity for viral clearance. In terms of *in vivo* viral load, all three treatments resulted in lower viral titers compared to the control group, with a trend of decreasing viral loads observed in the Bis-M1M3, bsAb A9F8-A3A1, and bsAb A9F8-B7C9 groups in that order (though no significant differences were noted) (Fig. 6D).

**Fig 7:**
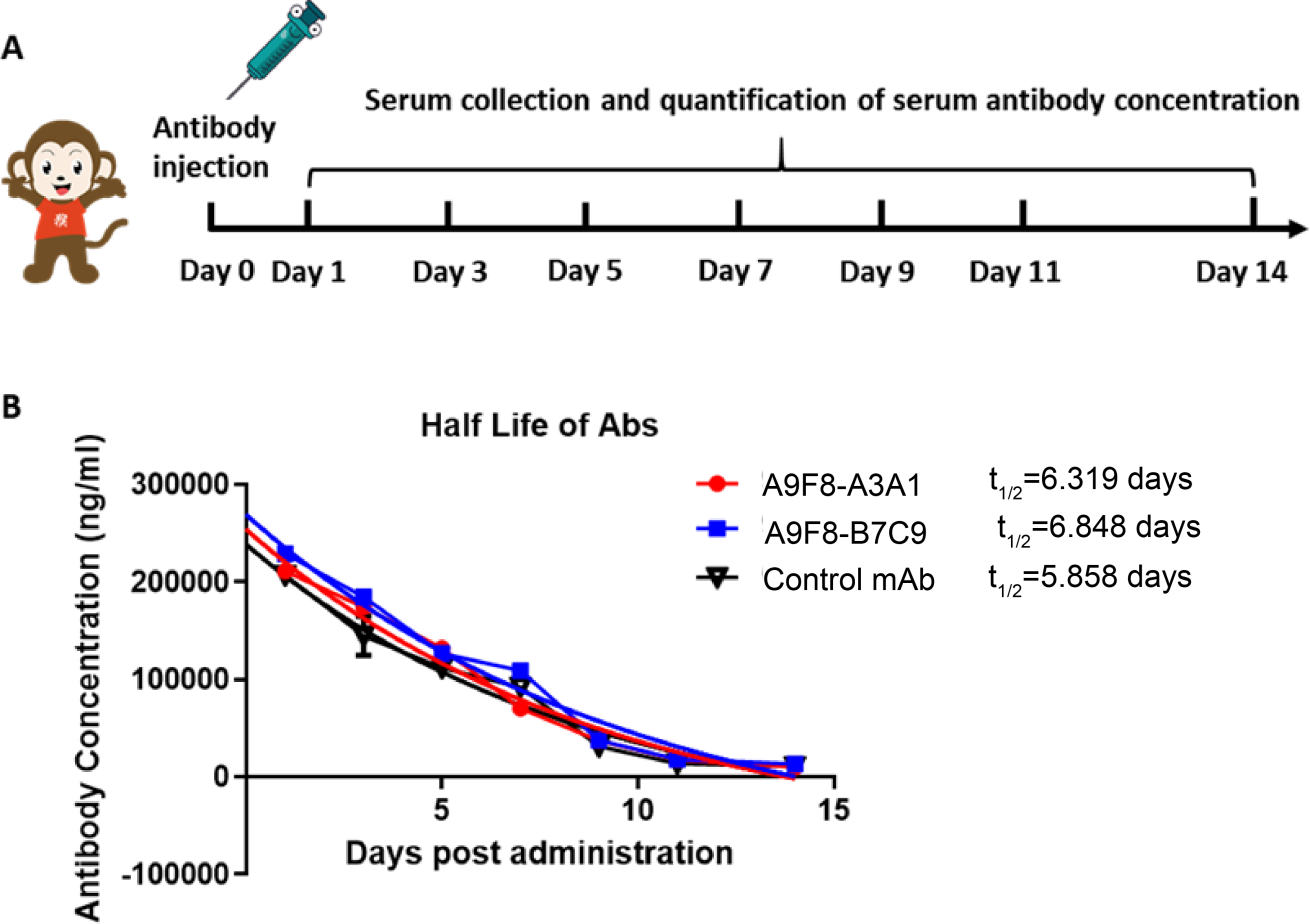
Serum antibody concentrations in rhesus macaques infused with chimeric antibodies (bsAb A9F8-A3A1 and bsAb A9F8-B7C9). (**A**) Schematic diagram indicating the antibody pharmacokinetics of bsAb A9F8-A3A1, bsAb A9F8-B7C9, and control antibody in rhesus monkeys. The rhesus macaques (*n* = 4 per group) were injected with each antibody in a dose of 10 mg/kg on day 0, and sera were collected on days 1, 3, 5, 7, 9, 11, and 14; the antibody concentration was determined by ELISA. (**B**) The half-life of each antibody in rhesus macaques was measured in serum over 14 days after the intravenous administration of each antibody.

Finally, to expand the clinical application potential of the bsAbs A9F8-A3A1 and A9F8-B7C9 against mpox, we determined the half-lives of these chimeric antibodies, which contain a human IgG1 constant region, in rhesus macaques. Subsequently, the antibodies were infused into the macaques, and their serum levels of the antibodies were analyzed over 14 days (Fig. 7A). The *in vivo* half-lives of bsAb A9F8-A3A1 and bsAb A9F8-B7C9 were 6.319 and 6.848 days, respectively, which was comparable to that of the control mAb (Fig. 7B).

## DISCUSSION

Infection with MPXV can cause serious complications and death in certain populations, including young children, pregnant women, and immunocompromised individuals (38–40). No specific therapeutic agents have been approved for monkeypox infection; current clinical management primarily focuses on three key aspects: management of secondary bacterial complications, provision of symptomatic relief, and implementation of supportive care measures (41, 42). Developing effective treatment methods is urgently needed.

Meanwhile, the preparation of monoclonal or antibody mixtures that can specifically recognize MPXV epitopes can be a safer and more effective way to treat monkeypox. Several monoclonal antibodies against poxviruses have been developed, showing high antiviral activity *in vivo* and *in vitro* (24, 43, 44), but the design of MPXV bsAbs has rarely been reported. It is worth noting that in our previous studies, we found that a cocktail of monoclonal antibodies had a more efficient anti-poxvirus effect *in vivo* and *in vitro* than a single antibody, possibly because the combination of antibodies can recognize different epitopes of MPXV, and the functional activity of the combination may exceed the total activity of the individual monoclonal antibodies, allowing for more efficient antiviral activity (45). However, the cost and complexity of combined therapy are higher than that of single therapy, so bsAbs have been developed, which have binding sites for two different epitopes or antigens, a more cost-effective strategy. More importantly, bsAbs have been used in clinical cancer treatment and are being developed for the treatment of various viral infections (46–48).

In this study, we designed bsAb A9F8-A3A1 to target two distinct non-overlapping binding epitopes of MPXV A29, and bsAb A9F8-B7C9 to target two different antigenic epitopes of MPXV A29 and MPXV B6, respectively. MPXV A29 is one of the membrane proteins on the IMV and is highly homologous to VACV A27. Research has shown that protective antibodies against A27 may interact with a binding site for acetylated heparin located adjacent to A27, potentially affecting ligand binding and thereby interfering with the functional activity of the antigen (18, 49). MPXV B6 is a membrane protein on the EEV and is highly homologous to VACV B5. Studies indicate that with the assistance of proteins such as B5, EEV can enter the cell following outer membrane rupture induced by contact with GAGs on the cell surface (50). Therefore, the design of bsAbs targeting distinct epitopes on both IMV and EEV could not only reduce the number of viral particles that have already entered the cell but also diminish viral transmission, thereby exerting a more effective antiviral effect. Initially, the *in vitro* neutralization results against the VACV (Tiantan) and VACV (WR) strains showed that bsAbs A9F8-B7C9 and A9F8-A3A1 had lower IC_50_ values compared to their parental antibodies, indicating *in vitro* antiviral effect. The higher IC_50_ values observed for various antibodies may be attributed to higher viral titers or different types of complement (baby rabbit complement or guinea pig serum) used. Based on these findings, MPXV neutralization assays were conducted, demonstrating strong neutralizing activity for all three bsAbs, with bsAb A9F8-B7C9 showing the best effect. This suggests that bispecific antibody designs targeting different viral antigen epitopes may harness the synergistic effects of each component more effectively and better prevent escape mutants. In terms of *in vivo* treatment, bsAbs provided complete protection against VACV Tiantan, VACV WR, and MPXV in mice, with no significant difference in therapeutic efficacy compared to Bis-M1M3, although bsAb A9F8-B7C9 tended to clear the virus from the body more rapidly. Further experiments will be conducted to explore whether bsAb A9F8-B7C9 possesses more efficient antiviral activity under various conditions. However, our study results indicate that bsAb A9F8-B7C9 has higher antiviral activity than its parental antibodies, similar efficacy to Bis-M1M3, and more significant *in vitro* neutralizing activity, suggesting that bsAb A9F8-B7C9 could be a highly potential therapeutic candidate. Moreover, its long half-life in crab-eating macaques makes it a promising candidate for subsequent clinical trials. It is noteworthy that due to experimental conditions, the current circulating MPXV clade I strain has not been obtained, and thus, its therapeutic effect against this strain has not been assessed. Further in-depth studies will continue. Additionally, this study adopted a symmetrical format for the designed dual antibody which retained the Fc segment and closely resembled natural antibodies; thus, it could be expressed more stably and was conducive to preservation (34). However, other forms of bsAbs such as asymmetric formats or fragment-based formats were not designed for comparison purposes; this represents one limitation of this study but also points toward future research directions. In the neutralization experiment, we utilized a hybrid virus comprising EEV and IMV. This viral form more closely mimics natural infection, as the natural infection process involves both EEV and IMV forms. However, precise quality control of the proportions of EEV and IMV in the hybrid virus is essential for ensuring the scientific rigor of the experiment. Currently, we have not regulated the quantities of EEV and IMV in the mix virus. In subsequent research efforts, we will endeavor to enhance our quality control measures in future experiments. Finally, this study primarily explored the antiviral activity of bispecific antibodies without conducting further studies on their antivirus mechanism. Cryo-electron microscopy can subsequently be utilized to characterize the binding epitopes of each component and antigens, thereby facilitating the development of diverse multi-specific antibodies.

## MATERIALS AND METHODS

### Mice and cells

Six- to eight-week-old female BALB/c mice were purchased from Shanghai Silaike Laboratory Animal Co., Ltd., and used for all experiments. The animals were maintained in individually ventilated cages and closely monitored for survival and signs of illness after the challenge. The guidelines for humane endpoints were strictly followed for all *in vivo* experiments. Animals that lost more than 25% of their initial body weight were immediately euthanized by CO_2_ asphyxiation and recorded as non-survivors.

BHK-21 cell lines were purchased from IMMOCELL (Xiamen, Fujian, China) and maintained in Dulbecco’s Modified Eagle’s Medium (DMEM) supplemented with 10% calf serum; 293 F cell lines were purchased from IMMOCELL and maintained in SMM 293-TII Expression Medium (Sino Biological).

### Viruses

VACV Tian Tan, VACV WR (ATCC VR-119), and MPXV (hMpxV/China/SZ-SZTH41/2023, EPI_ISL_18213375) were propagated and titrated in monolayer cultures of BHK-21 cells.

#### EEV preparations

As previously described, T75 flasks containing BHK cells at a 90% confluence were washed with phosphate-buffered saline (PBS), followed by the addition of VACV at a multiplicity of infection (MOI) of 0.5 for viral infection. After 48 h, the cell supernatant was collected and centrifuged at 4°C for 8 min at 450 × *g* to remove cellular debris. The supernatant was then stored at 4°C for subsequent preparation. The integrity and density of the EEV outer film were observed using transmission electron microscopy (JEOL-1400). A 10 µL sample was placed on a copper mesh (D&B) under a transmission electron microscope. After 10 min, phosphotungstic acid (D&B) was added, and staining was performed for 30 s. The sample was allowed to dry thoroughly before examination. However, only the EEV size and sample density could be observed, while the integrity of the membrane could not be clearly observed (Fig. S6). (20)

#### IMV preparations

In a similar manner, VACV preparations were infected at an MOI of 0.5 (20). After 72 h post-infection, the virus stock was harvested by subjecting the culture to three freeze-thaw cycles. IMV was purified using a sucrose gradient. The harvested virus was first subjected to ultrasound treatment for 40 s, then resuspended in a buffer containing 36% sucrose and centrifuged in an SW28 rotor at 13,500 rpm (33,000 × *g*) at 4°C for 80 min. The resulting precipitate was resuspended in 1× PBS and stored at −80°C until use.

#### Mixed IMV/EEV

To generate mixed IMV/EEV populations, cells were infected at an MOI of 0.5 and monitored for cytopathic effects (CPE) over 72 h. When plaque formation covered ≥50% of the culture surface, infected cells were harvested using a sterile cell scraper. The cell suspension was centrifuged at 2,000 × *g* for 2 min at 4°C, and the pellet was subjected to three freeze-thaw cycles (−80°C/37°C) to lyse cells and release intracellular virions. The lysate was combined with the initial supernatant and clarified by centrifugation (2,000 × *g* for 10 min at 4°C) to remove cellular debris. The resulting supernatant, containing mixed IMV and EEV particles, was briefly stored at 4°C and used immediately for downstream assays.

#### Viral titration

Initially, BHK-21 cells were seeded into 96-well plates at a density of 5 × 10^3^ cells per well and incubated for 24 h to form a confluent monolayer. Thereafter, the virus was serially diluted with 14 gradient steps, with five replicates per dilution, and 35 µL of each dilution was added to the prepared BHK-21 cells. The plates were incubated in a 5% CO_2_ incubator at 37°C for 1 h, followed by the addition of 200 µL of DMEM supplemented with 2% fetal bovine serum (FBS). For the EEV assay, the above steps were performed after incubation with neutralizing mAb M1H11 (32). On the fourth day post-inoculation, the CPE was examined microscopically, and the viral titer, expressed as TCID_50_, was ascertained using the Reed-Muench method.

### Construction and expression of chimeric antibody and bispecific antibody

#### Construction and expression of A9F8, B7C9, and A3A1

In accordance with the methodology outlined in a previous experiment (31), monoclonal antibodies 9F8, 3A1, and 7C9 were incorporated into the constant region of human IgG1 HC or kappa LC using overlapping extended PCR. Each antibody’s HC and LC were fused with signal peptides (METDTLLLWVLLLWVPGSTGD) and inserted into pTT5 at EcoR I/BamH I. Plasmid DNA was amplified in DH5α (Beijing Tsingke Biotech Co., Ltd; TSC-C01) and purified using a TIANQuick Midi Purification Kit (TIANGEN, China). The recombinant antibody was then transferred to the 293F cell line and purified using protein A columns (BBI, China).

#### Construction and expression of A9F8-B7C9 and A9F8-A3A1

The heavy-chain variable region of 7C9 and 3A1 is fused with the constant region of human IgG1 H. Additionally, the Fc end of the variable region is linked to the ScFv structure of 9F8 via a linker sequence (G4S)3. Each antibody’s HC and LC were fused with signal peptides (METDTLLLWVLLLWVPGSTGD) before being inserted into pTT5 at EcoR I/BamH I. Plasmid DNA amplification took place in DH5α cells from Beijing Tsingke Biotech Co., Ltd; TSC-C01 followed by purification using a TIANQuick Midi Purification Kit from TIANGEN. Subsequently, the recombinant antibodies were introduced to the 293 F cell line for further purification utilizing protein A columns (BBI).

### SDS-PAGE analysis of the purified antibodies

The purified recombinant antibodies were separated using 12% SDS-PAGE (Omni-Easy, EpiZyme), stained with Coomassie blue for 10 min, then decolorized with pure water on a shaking table, and the results were observed and analyzed.

### ELISA and SPR

#### The interaction between antigens and antibodies was assessed using an ELISA

Microtiter plates (WANTAI BioPharm) were coated with 5 µg/mL of purified proteins (50 µL per well) overnight at 4°C. After three washes, the plates were sealed with a 2% wt/vol skim milk powder in PBS solution and incubated at 37°C for 2 h. Following another round of washing and drying, diluted antibodies were added to each well and incubated at 37°C for 30 min. Subsequently, a solution of horseradish peroxidase (HRP)-coupled goat anti-mouse IgG antibody was added to each well, followed by a further incubation at 37°C for 30 min. After five washes, tetramethylbenzidine substrate (WANTAI BioPharm) was added to each well at room temperature in the dark. The reaction was stopped after 15 min with a solution of 2 M H_2_SO_4_, and the absorbance was measured at 450 nm. All samples were analyzed in triplicate. The relative affinity of antibody binding to purified viruses or proteins was determined by measuring the concentration required to achieve the EC50.

#### The dissociation constant of antibody-antigen binding was evaluated using SPR

A pre-prepared Dextran chip was used, into which protein samples were injected quickly into the sample area at a volume of 0.1 µL before being left overnight at 4°C on a shaking bed. The chips were then washed with PBS three times before adding a solution of ethanolamine aqueous solution and allowing it to react for half an hour away from light. Following another round of washing with PBS and blowing dry with nitrogen gas, the chips were covered before loading flow phase samples (each sample volume being set as 700 µL) into PlexArray HT A100 after centrifuging diluted antibodies beforehand at low-temperature conditions (4°C, at 12,000 rpm for 15 min). The flow phase samples were sequentially sampled from low concentration to high concentration at a rate of 2 µL/s, and the data were collected and analyzed.

#### Immunofluorescence

Immunofluorescence was performed on the VACV Tiantan strain (donated by Yang Yang, The Third People’s Hospital of Shenzhen, Shenzhen, China). In the co-incubation group, the virus, antibody, and 5% guinea pig serum were co-incubated for 1 h. Subsequently, the mixture was added to the cells and incubated for an additional 2 h. The supernatant was removed, and fresh medium was added. In the pre-infection group, cells were first infected with the virus for 1 h, then washed with PBS. Then, antibodies containing 5% guinea pig serum were added. After a 12 h culture period, the cells were fixed with 4% paraformaldehyde and washed three times with PBS. Subsequently, 0.5% Triton X-100 was used for permeabilization in the dark for 30 min. After the removal of the permeabilization solution, the cells were blocked in the dark for another 30 min. Following the removal of the blocking solution, the primary antibody was added, and the samples were incubated at 4°C overnight. After three washes with PBS, the secondary antibody was introduced and incubated at room temperature in the dark for 30 min. Subsequently, the 4′,6-diamidino-2-phenylindole (DAPI) dye solution was added and incubated at room temperature in the dark for 5 min. Finally, the samples were washed three times with PBS under dark conditions. Anti-fluorescence quenching and mounting medium were then applied. An inverted confocal laser microscope (FV3000) was utilized for observation and imaging. Green fluorescence with an excitation wavelength of 495 nm and an emission wavelength of 519 nm was selected for detection.

#### Microneutralization assay

To evaluate the neutralizing activity of antibodies, microneutralization experiments of EEV, IMV, and mixed viruses were performed. Among them, when the neutralization experiment was determined using EEV, the neutralizing monoclonal antibody, M1H11, which targets M1, was added and incubated for 1 h to consume the IMV therein. Its titer was then determined by the standard plaque assay (Fig. S7). Initially, the purified antibodies are serially diluted in a 96-well plate, and the negative control group was replaced with 2% FBS + DMEM, followed by the addition of an equal molar amount of virus at a titer of 100 TCID_50_ or 200 PFU, along with 5% complement from baby rabbits (Pel-Freez Biologicals). The mixture was then incubated at 37°C with 5% CO_2_ for 1 h. Subsequently, the BHK cells are prepared, washed with 1× PBS, and exposed to 35 µL of the pre-incubated virus mixture. After a 2 h infection, the supernatant was removed, and the CPE was assessed on the fourth day post-infection. The IC_50_ value was determined using GraphPad Prism software.

#### Comet inhibition assay

The neutralization activity of each antibody was evaluated *in vitro* using the comet inhibition assay. BHK-21 cells were seeded onto six-well plates 1 day previously, following the established protocol (51), and incubated until a confluent monolayer was achieved for subsequent use. Subsequently, 50 PFU of VACV Tiantan was added. After a 2 h incubation at 37°C, the cells were washed twice with PBS and then supplemented with fresh medium containing 60 µg of antibody. The PBS-treated group served as the negative control. Forty-eight hours later, the cells were stained with crystal violet, and the results were visualized and photographed.

#### Therapeutic efficacy studies in mice

Female mice aged between 6 and 8 weeks were randomly allocated into seven groups consisting of seven mice per group. Following anesthesia using isoflurane and oxygen, the mice received intranasal administration of VACV Tiantan/VACV WR and MPXV at a dosage of 1 × 10^6^ TCID50. Additionally, antibodies at a concentration of 5 mg/kg were administered via intraperitoneal injection 24 h post-infection. Subsequently, on the fourth day post-infection, lung samples from three randomly chosen individuals underwent viral titration and pathological analysis. The weights and conditions of the mice were monitored continuously for 10 days.

### Reverse transcription PCR and tissue staining

#### Reverse transcription PCR detection of viral load in lung tissue

The lung tissue samples collected were separated, ground, and suspended in 1,000 µL 1× PBS. The samples were homogenized using a cryogenic grinder (LUKYM-I, 70 Hz, 5 min) and centrifuged to obtain the supernatant. The nucleic acid was extracted using the FastPure Viral DNA/RNA Mini Kit Pro (Vazyme, China), and then the viral load was measured by reverse transcription PCR experiment. The primer sequences for qPCR were as follows: vaccinia virus forward, 5′-ACATCTGGAGAATCCACAACA-3′; vaccinia virus reverse, 5′-CATCATCGGTGGTTGATTTA-3′; and vaccinia virus probe, 5′-FAM-GAGACTCCGGAACCAAT-TAMRA-3′. All samples were run in triplicate. The results were analyzed and plotted using GraphPad version 9.0 software.

#### Tissue staining was used to analyze lung lesions

After fixing the collected lung specimens in 4% paraformaldehyde for 48 h, they were stained with hematoxylin and eosin for histopathological evaluation. The stained sections were analyzed under a Nikon 80i microscope with ×10 and ×40 objectives and examined by pathologists.

#### Half-life quantitation

Eight male rhesus macaques were housed and cared for in a biosafety level 2 facility. Four macaques were administered intravenously with low-endotoxin antibody at 10 mg/kg, and sera were collected before and at various time points after injection. Antibody concentrations in serum were measured by quantitative ELISA. A29-coated microtiter plates were used to capture the antibody followed by detection using an HRP-conjugated anti-human IgG antibody. The other four macaques were injected with an equivalent dose of an influenza antibody as a control. Pharmacokinetic parameters were calculated in WinNonlin Software using the non-compartment model.

### Statistical analysis

The data are presented as the mean ± standard deviation from a minimum of three independent experiments. Group differences were analyzed using paired Student’s *t*-test and one-way analysis of variance, followed by Tukey’s post hoc test (GraphPad Prism version 9.0). Statistical significance was set at *P* < 0.05.

## Data Availability

All data needed to evaluate the conclusions in the paper are present in the paper and the supplemental material.

## SUPPLEMENTAL MATERIAL

Supplemental material jvi.02320-24-s0001.docx Figures S1 to S7 and Table S1.

ASM does not own the copyrights to Supplemental Material that may be linked to, or accessed through, an article. The authors have granted ASM a non-exclusive, world-wide license to publish the Supplemental Material files. Please contact the corresponding author directly for reuse.
