# Erratum for Li et al., “Bispecific antibodies targeting MPXV A29 and B6 demonstrate efficacy against MPXV infection”

**DOI:** 10.1128/jvi.00350-26

**Published:** 2026-03-16

**Authors:** Mengjun Li, Jiayin Chen, Fuxiang Wang, Jiahua Kuang, Yun Peng, Sadia Asghar, Wei Zhao, Yang Yang, Chenguang Shen

## ERRATUM

Volume 99, no. 5, e02320-24, 2025, https://doi.org/10.1128/jvi.02320-24. Fuxiang Wang’s name should appear as shown in this erratum.

