# Supplementary material for: Bispecific antibodies targeting MPXV A29 and B6 demonstrate efficacy against MPXV infection
Source: J Virol. 2025 Apr 3;99(5):e02320-24. doi: 10.1128/jvi.02320-24 (PMC12090737; doi:10.1128/jvi.02320-24)
Supplement: Supplemental material — Figures S1 to S7 and Table S1. [file jvi.02320-24-s0001.docx]

**Bispecific Antibodies Targeting MPXV A29 and B6 Demonstrate Efficacy Against MPXV Infection**

Mengjun Li^1‡^, Jiayin Chen^1^^‡^, Fuxiang Wang^2‡^, Jiahua Kuang^2‡^, Yun Peng^2^, Sadia Asghar^1^, Wei Zhao^1^*, Yang Yang^2^*, Chenguang Shen^1,3,4^*

^1^BSL-3 Laboratory (Guangdong), Guangdong Provincial Key Laboratory of Tropical Disease Research, School of Public Health, Southern Medical University, Guangzhou, People's Republic of China.

^2^National Clinical Research Center for infectious disease, Shenzhen Third People's Hospital, Second Hospital Affiliated to Southern University of Science and Technology, 518112 Shenzhen, People’s Republic of China.

^3^Department of Laboratory Medicine, Zhujiang Hospital; Southern Medical University, Guangzhou, People's Republic of China.

^4^Key Laboratory of Infectious Diseases Research in South China (Southern Medical University), Ministry of Education, Guangzhou, Guangdong, 510515, China.

*Corresponding author. Email: [a124965468@smu.edu.cn](mailto:a124965468@smu.edu.cn) (C.S), [young@mail.sustech.edu.cn](mailto:young@mail.sustech.edu.cn) (Y.Y) and [zhaowei@smu.edu.cn](mailto:zhaowei@smu.edu.cn) (W.Z)

^‡^These authors contributed equally to this work.


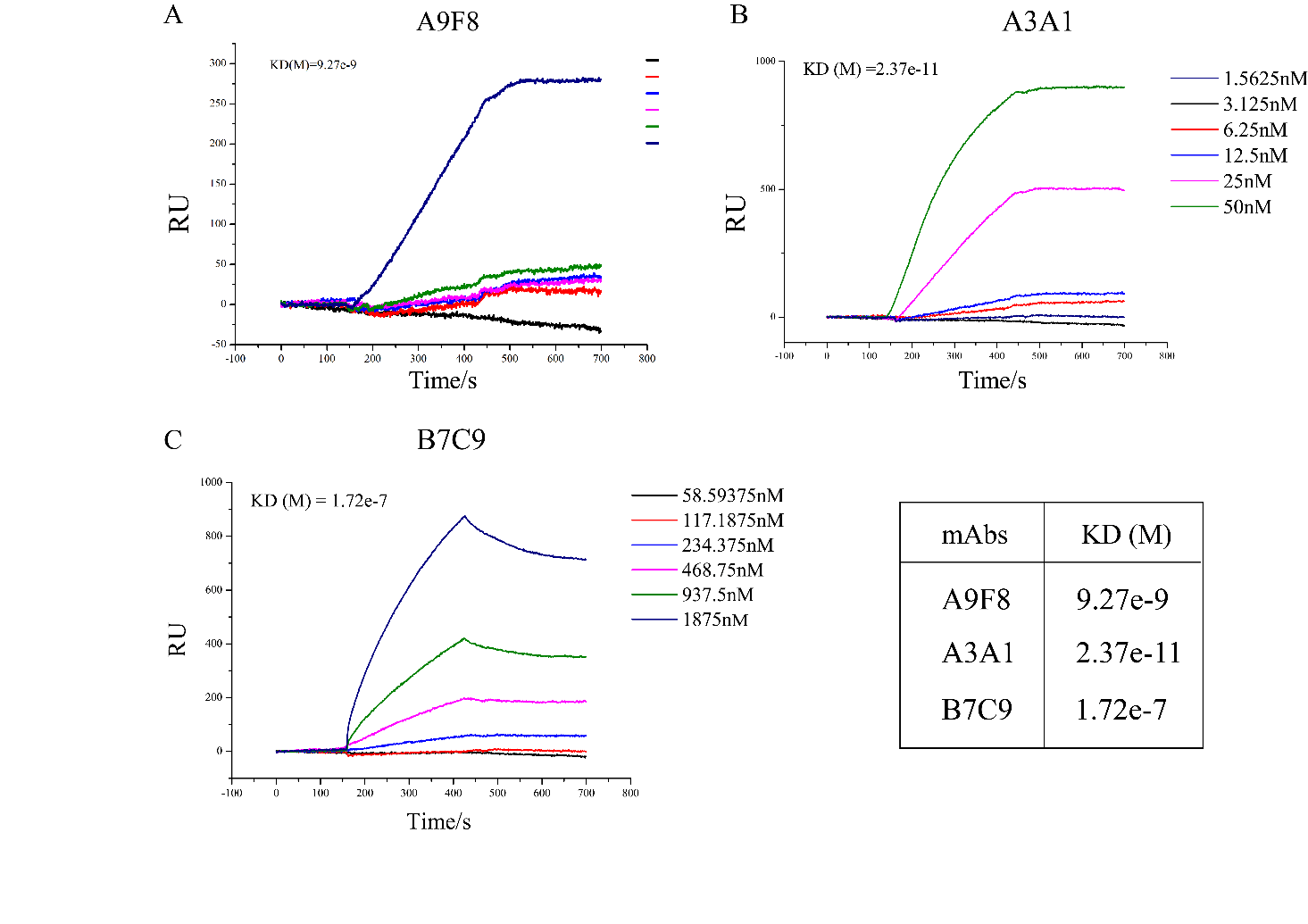


**Fig S1.** Binding affinity analysis of mAbs to MPXV A29L and MPXV B6R. (A) Affinity results for humanized mAb C9F8 to MPXV A29L. (B) Affinity results for mAb C3A1 aganist MPXV B6R. (C) Affinity results of the humanized mAb C7C9 to MPXV A29L.


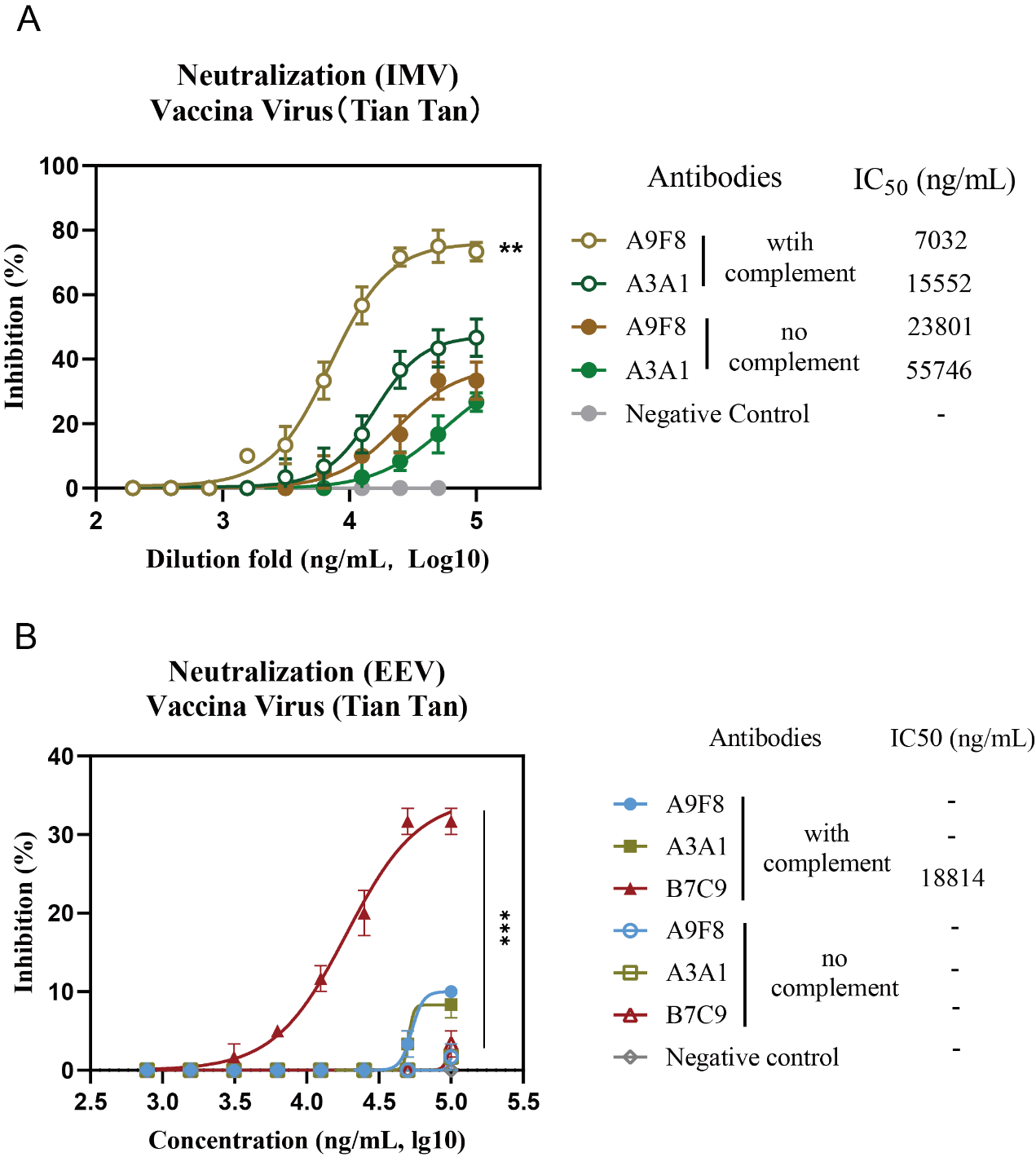


**Fig S2.** Neutralization of VACV (Tiantan) IMV/EEV by Humanized Monoclonal Antibodies A9F8, A3A1 and B7C9 in the Presence or Absence of Guinea Pig Serum as a Complement Source. (A) Neutralization of VACV (Tiantan) IMV by A9F8, A3A1 in the Presence or Absence of Guinea Pig Serum as a Complement Source. (B) Neutralization of VACV (Tiantan) EEV by A9F8, A3A1 and B7C9 in the Presence or Absence of Guinea Pig Serum as a Complement Source. Statistical significance was calculated *via* ordinary one-way ANOVA. *p < 0.05, **p < 0.01, ***p < 0.001.


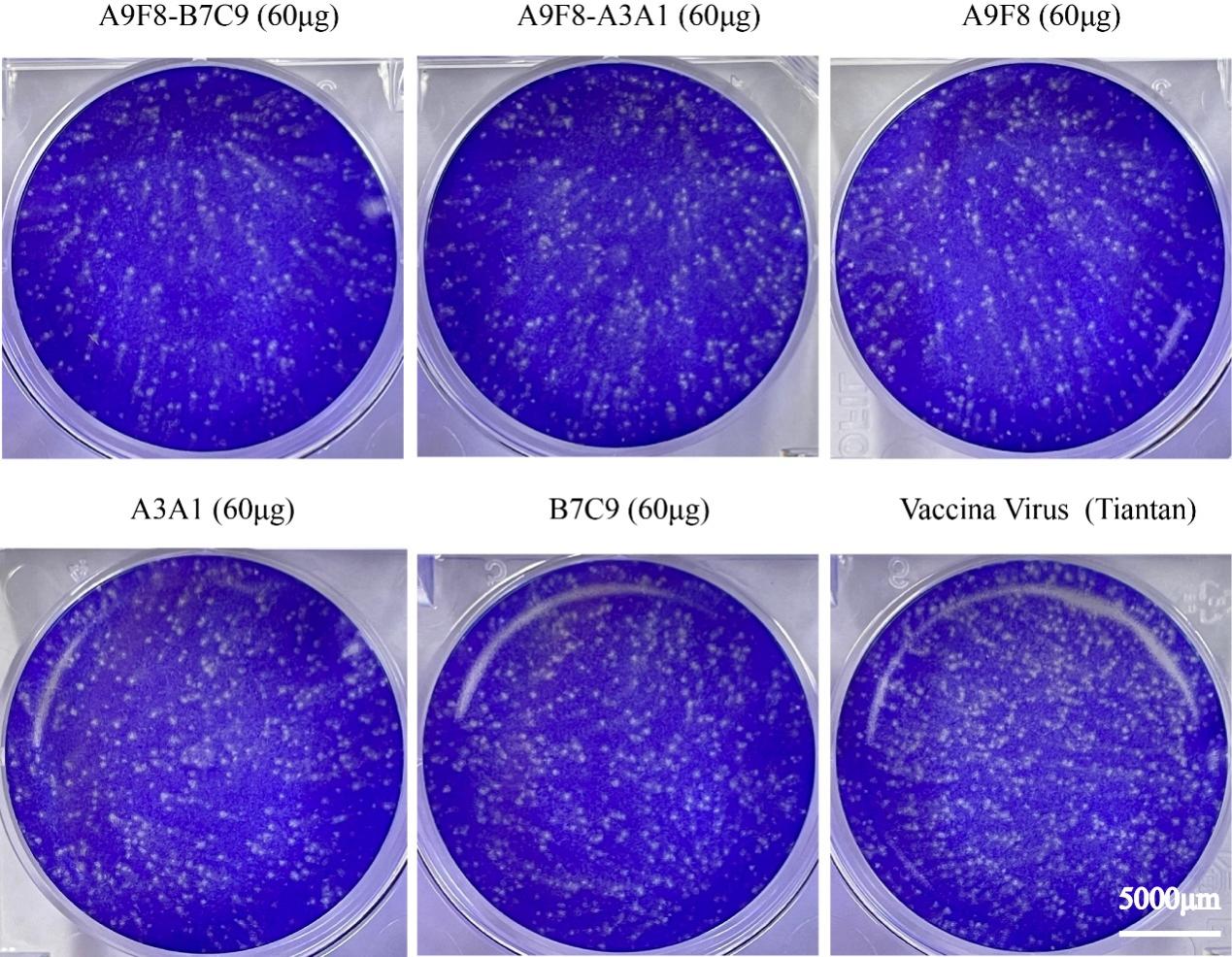


**Fig S3.** Activity of anti-B6 monoclonal antibody C7C9 was determined in vitro by comet inhibition assay. Monolayer BHK cells were infected with 50PFU VACV Tiantan for 2h at 37 ℃, washed and supplemented with medium containing antibodies. Fresh medium was used as a negative control.


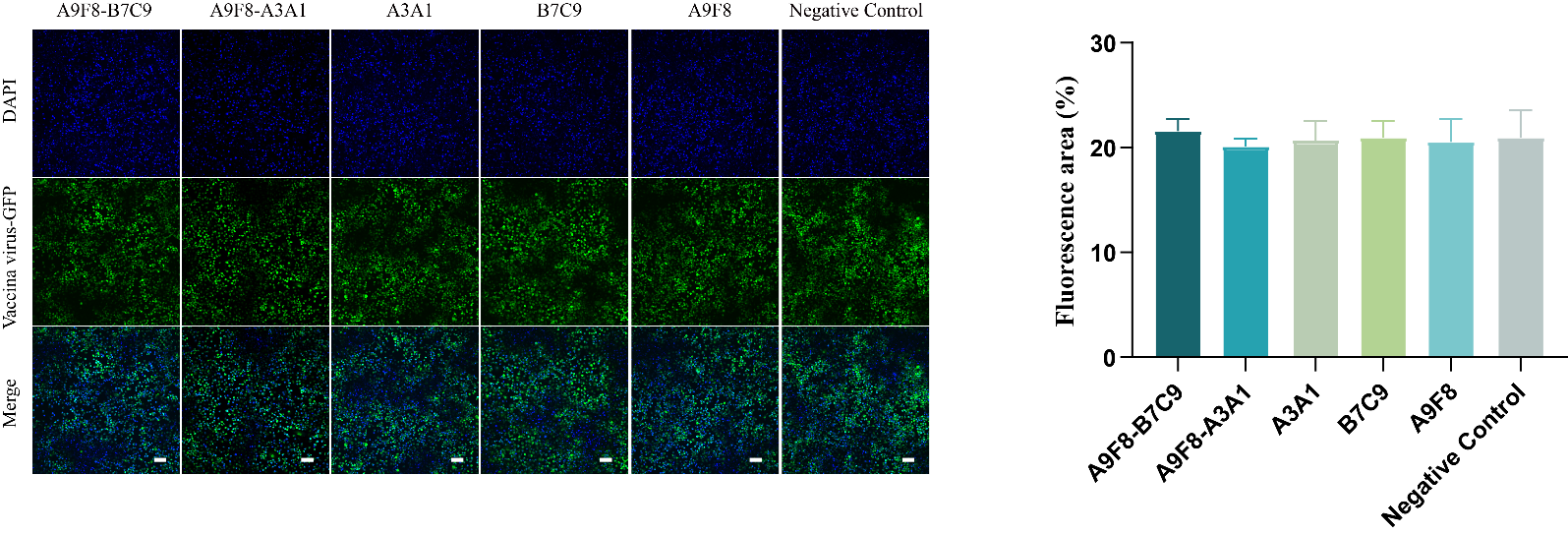


**Fig S4. Confocal images of mAbs and bsAbs immunofluorescence experiments.** The cells were infected with virus for 1h, and then incubated with the same volume of antibody for 2h. ImageJ was used to quantitatively analyze the proportion of fluorescent area in each treatment group. Performed in the presence of 5% complement. Scale bars, 100μm. Data were displayed as mean ± SD (n = 3). Statistical significance was calculated via ordinary one-way ANOVA.


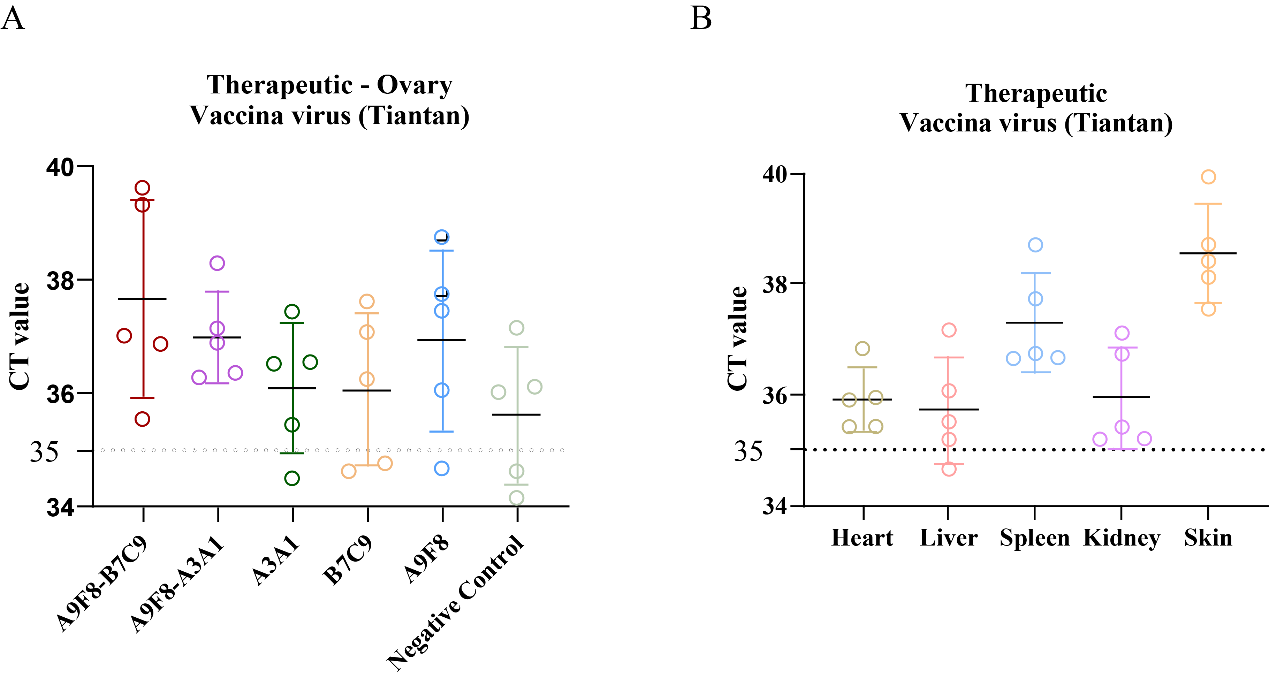


**Fig S5. Therapeutic efficacy of bsAbs in a mouse model of VACV (Tiantan).** (A) Ovary virus titer was detected by RT-PCR. (B) Viral load in all organs (heart, liver, spleen, kidney, skin) of Control IgG group was detected by RT-PCR. All data are shown as means with SD and were analyzed by one-way analysis of variance.


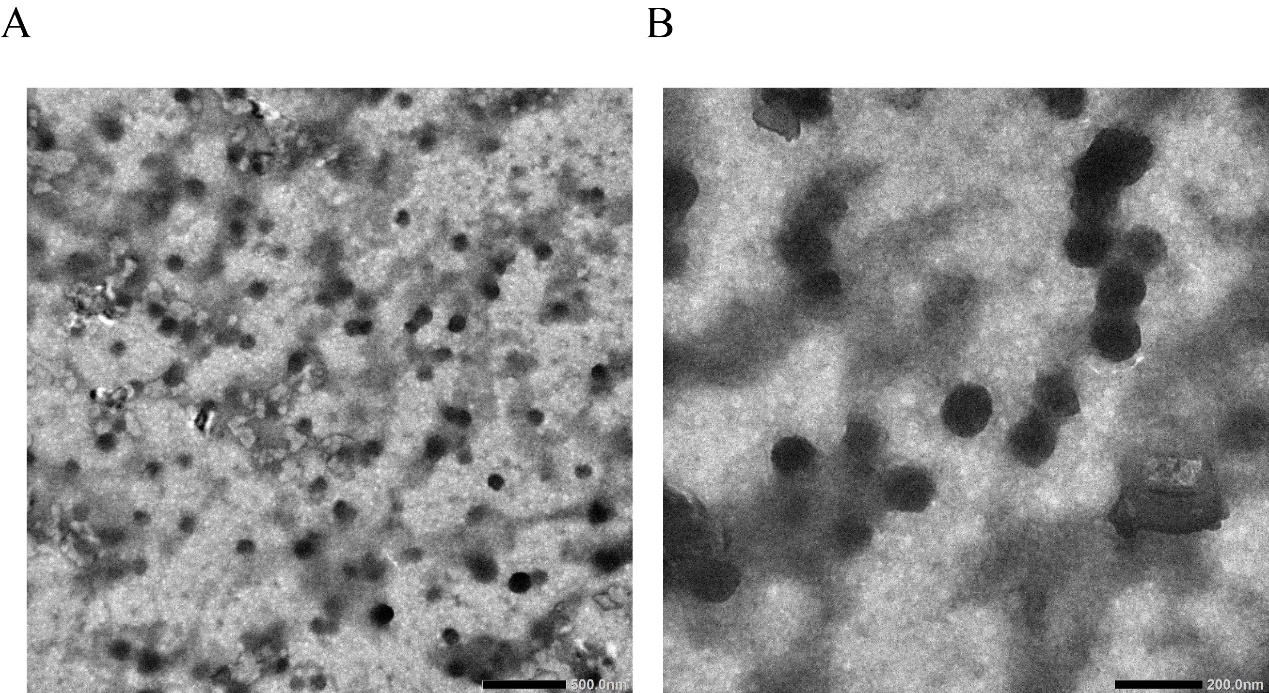


Fig S6. Transmission electron microscopy, fresh EEV density, and outer membrane integrity. (A-B) EEV particles observed in different field of view, scale =500μm (A) /200μm (B).


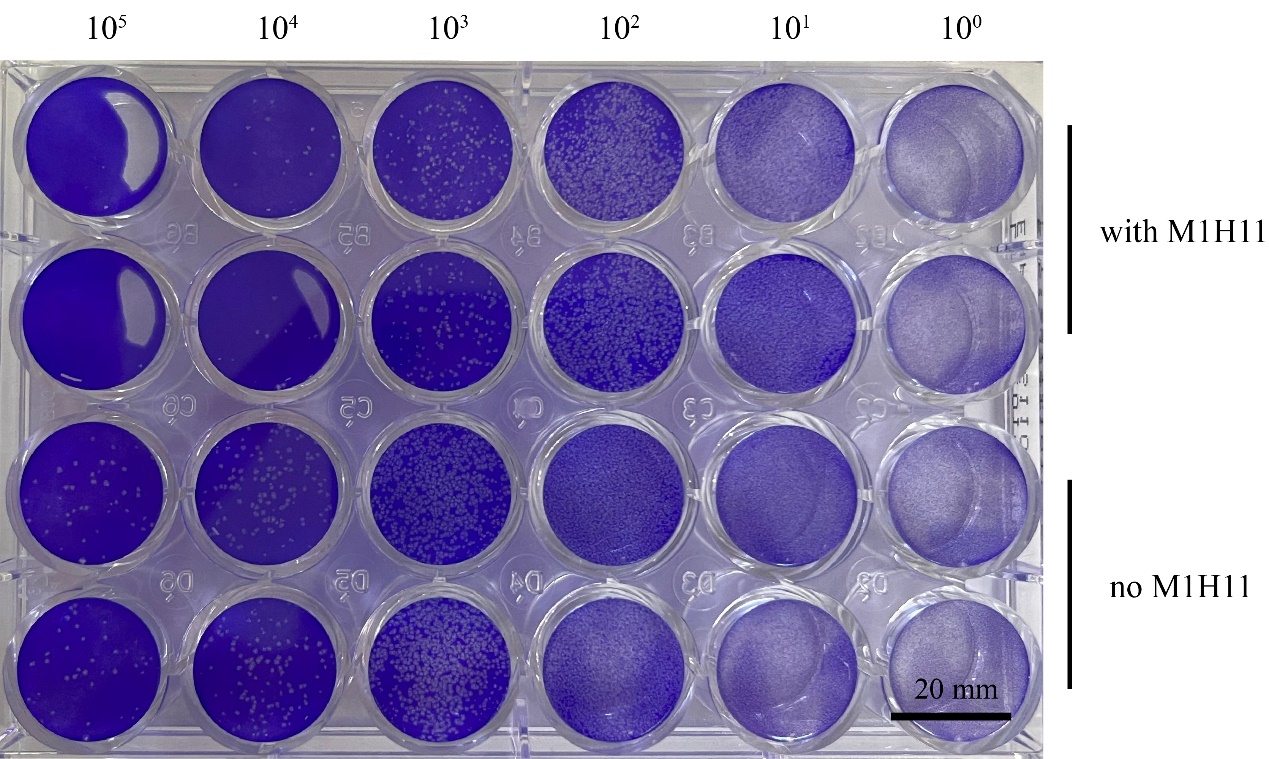


Fig S7. The titer of extracellular enveloped virus (EEV) was determined using a standard plaque assay. The samples were divided into two groups to compare the titers of M1H11 before and after incubation, with two replicates in each group.

Table S1. Recombinant antibody sequences.

| antibodies | Heavy chain | Light chain |
| --- | --- | --- |
| A9F8-B7C9 | METDTLLLWVLLLWVPGSTGDEVKLLESGGGLVQPGGSLKLSCAASGFDFSRYWMSWVRQAPGKGLEWIGEINPDSSTINYTPSLKDKFIISRDNAKNTLYLQMSKVRSEDTALYYCSRRGANWGQGTLVTVSSASTKGPSVFPLAPSSKSTSGGTAALGCLVKDYFPEPVTVSWNSGALTSGVHTFPAVLQSSGLYSLSSVVTVPSSSLGTQTYICNVNHKPSNTKVDKRVEPKSCDKTHTCPPCPAPELLGGPSVFLFPPKPKDTLMISRTPEVTCVVVDVSHEDPEVKFNWYVDGVEVHNAKTKPREEQYNSTYRVVSVLTVLHQDWLNGKEYKCKVSNKALPAPIEKTISKAKGQPREPQVYTLPPSRDELTKNQVSLTCLVKGFYPSDIAVEWESNGQPENNYKTTPPVLDSDGSFFLYSKLTVDKSRWQQGNVFSCSVMHEALHNHYTQKSLSLSPGKGGGGSGGGGSGGGGSGGGGSGGGGSQVQLQQPGAELVKPGASVKLSCKASGYTFTSYWMHWVKQRPGQGLEWIGEINPSNGRTNYNEKFKSKATLTVDKSSSTAYMQLSSLTSEDSAVYYCARSDRYDDFAYWGQGTLVTVSAGGGGSGGGGSGGGGSDVVMTQTPLSLPVSLGDQASISCRSSQSLVHSNGNTYLHWYLQKPGQSPKLLIYKVSNRFSGVPDRFSGSGSGTDFTLKISRVEAEDLGVYFCSQSTHVPLTFGAGTKLELK* | METDTLLLWVLLLWVPGSTGDDVVMTQTPLSLSVTIGQPASISCKSSQSLLYSNGKTYLNWLQQRPGQAPKHLMYQVSKLDPGIPDRFSGSGSETDFTLKISRVEAEDLGVYYCLQGTYYPHTFGGGTKLEIKRTVAAPSVFIFPPSDEQLKSGTASVVCLLNNFYPREAKVQWKVDNALQSGNSQESVTEQDSKDSTYSLSSTLTLSKADYEKHKVYACEVTHQGLSSPVTKSFNRGECS* |
| A9F8-A3A1 | METDTLLLWVLLLWVPGSTGDEVKLLESGGGLVQPGGSLKLSCAASGFDFSRYWMSWVRQAPGKGLEWIGEINPDSSTINYTPSLKDKFIISRDNAKNTLYLQMSKVRSEDTALYYCSRRGANWGQGTLVTVSSASTKGPSVFPLAPSSKSTSGGTAALGCLVKDYFPEPVTVSWNSGALTSGVHTFPAVLQSSGLYSLSSVVTVPSSSLGTQTYICNVNHKPSNTKVDKRVEPKSCDKTHTCPPCPAPELLGGPSVFLFPPKPKDTLMISRTPEVTCVVVDVSHEDPEVKFNWYVDGVEVHNAKTKPREEQYNSTYRVVSVLTVLHQDWLNGKEYKCKVSNKALPAPIEKTISKAKGQPREPQVYTLPPSRDELTKNQVSLTCLVKGFYPSDIAVEWESNGQPENNYKTTPPVLDSDGSFFLYSKLTVDKSRWQQGNVFSCSVMHEALHNHYTQKSLSLSPGKGGGGSGGGGSGGGGSGGGGSGGGGSEVKLVESGGGLVQPGGSLRLSCATSGFTFSDFYMEWVRQPPGKRLEWIAASRNKANDYTTKYSASVKGRFNVSRDTSQSILYLQMNALRAEDSAIYYCARGYRFDGAWFAYWGQGTLVTVSAGGGGSGGGGSGGGGSDIVMTQSHKFMSTSVGDRVSITCKASQDVGTEVAWYQQKPGQSPKLLIYWASTRHTGVPDRFTGSGSGTDFTLTISNVQSEDLADYFCQQYSSYPLTFGSGTKLEIK* | METDTLLLWVLLLWVPGSTGDDVVMTQTPLSLSVTIGQPASISCKSSQSLLYSNGKTYLNWLQQRPGQAPKHLMYQVSKLDPGIPDRFSGSGSETDFTLKISRVEAEDLGVYYCLQGTYYPHTFGGGTKLEIKRTVAAPSVFIFPPSDEQLKSGTASVVCLLNNFYPREAKVQWKVDNALQSGNSQESVTEQDSKDSTYSLSSTLTLSKADYEKHKVYACEVTHQGLSSPVTKSFNRGECS* |
| A9F8 | METDTLLLWVLLLWVPGSTGDEVKLLESGGGLVQPGGSLKLSCAASGFDFSRYWMSWVRQAPGKGLEWIGEINPDSSTINYTPSLKDKFIISRDNAKNTLYLQMSKVRSEDTALYYCSRRGANWGQGTLVTVSSASTKGPSVFPLAPSSKSTSGGTAALGCLVKDYFPEPVTVSWNSGALTSGVHTFPAVLQSSGLYSLSSVVTVPSSSLGTQTYICNVNHKPSNTKVDKRVEPKSCDKTHTCPPCPAPELLGGPSVFLFPPKPKDTLMISRTPEVTCVVVDVSHEDPEVKFNWYVDGVEVHNAKTKPREEQYNSTYRVVSVLTVLHQDWLNGKEYKCKVSNKALPAPIEKTISKAKGQPREPQVYTLPPSRDELTKNQVSLTCLVKGFYPSDIAVEWESNGQPENNYKTTPPVLDSDGSFFLYSKLTVDKSRWQQGNVFSCSVMHEALHNHYTQKSLSLSPGK* | METDTLLLWVLLLWVPGSTGDDVVMTQTPLSLSVTIGQPASISCKSSQSLLYSNGKTYLNWLQQRPGQAPKHLMYQVSKLDPGIPDRFSGSGSETDFTLKISRVEAEDLGVYYCLQGTYYPHTFGGGTKLEIKRTVAAPSVFIFPPSDEQLKSGTASVVCLLNNFYPREAKVQWKVDNALQSGNSQESVTEQDSKDSTYSLSSTLTLSKADYEKHKVYACEVTHQGLSSPVTKSFNRGECS* |
| A3A1 | METDTLLLWVLLLWVPGSTGDEVKLVESGGGLVQPGGSLRLSCATSGFTFSDFYMEWVRQPPGKRLEWIAASRNKANDYTTKYSASVKGRFNVSRDTSQSILYLQMNALRAEDSAIYYCARGYRFDGAWFAYWGQGTLVTVSAASTKGPSVFPLAPSSKSTSGGTAALGCLVKDYFPEPVTVSWNSGALTSGVHTFPAVLQSSGLYSLSSVVTVPSSSLGTQTYICNVNHKPSNTKVDKRVEPKSCDKTHTCPPCPAPELLGGPSVFLFPPKPKDTLMISRTPEVTCVVVDVSHEDPEVKFNWYVDGVEVHNAKTKPREEQYNSTYRVVSVLTVLHQDWLNGKEYKCKVSNKALPAPIEKTISKAKGQPREPQVYTLPPSRDELTKNQVSLTCLVKGFYPSDIAVEWESNGQPENNYKTTPPVLDSDGSFFLYSKLTVDKSRWQQGNVFSCSVMHEALHNHYTQKSLSLSPGK* | METDTLLLWVLLLWVPGSTGDDIVMTQSHKFMSTSVGDRVSITCKASQDVGTEVAWYQQKPGQSPKLLIYWASTRHTGVPDRFTGSGSGTDFTLTISNVQSEDLADYFCQQYSSYPLTFGSGTKLEIKRTVAAPSVFIFPPSDEQLKSGTASVVCLLNNFYPREAKVQWKVDNALQSGNSQESVTEQDSKDSTYSLSSTLTLSKADYEKHKVYACEVTHQGLSSPVTKSFNRGECS* |
| B7C9 | METDTLLLWVLLLWVPGSTGDQVQLQQPGAELVKPGASVKLSCKASGYTFTSYWMHWVKQRPGQGLEWIGEINPSNGRTNYNEKFKSKATLTVDKSSSTAYMQLSSLTSEDSAVYYCARSDRYDDFAYWGQGTLVTVSAASTKGPSVFPLAPSSKSTSGGTAALGCLVKDYFPEPVTVSWNSGALTSGVHTFPAVLQSSGLYSLSSVVTVPSSSLGTQTYICNVNHKPSNTKVDKRVEPKSCDKTHTCPPCPAPELLGGPSVFLFPPKPKDTLMISRTPEVTCVVVDVSHEDPEVKFNWYVDGVEVHNAKTKPREEQYNSTYRVVSVLTVLHQDWLNGKEYKCKVSNKALPAPIEKTISKAKGQPREPQVYTLPPSRDELTKNQVSLTCLVKGFYPSDIAVEWESNGQPENNYKTTPPVLDSDGSFFLYSKLTVDKSRWQQGNVFSCSVMHEALHNHYTQKSLSLSPGK* | METDTLLLWVLLLWVPGSTGDDVVMTQTPLSLPVSLGDQASISCRSSQSLVHSNGNTYLHWYLQKPGQSPKLLIYKVSNRFSGVPDRFSGSGSGTDFTLKISRVEAEDLGVYFCSQSTHVPLTFGAGTKLELKRTVAAPSVFIFPPSDEQLKSGTASVVCLLNNFYPREAKVQWKVDNALQSGNSQESVTEQDSKDSTYSLSSTLTLSKADYEKHKVYACEVTHQGLSSPVTKSFNRGECS* |
